# Inter-comparison of multiple statistically downscaled climate datasets for the Pacific Northwest, USA

**DOI:** 10.1038/sdata.2018.16

**Published:** 2018-02-20

**Authors:** Yueyang Jiang, John B. Kim, Christopher J. Still, Becky K. Kerns, Jeffrey D. Kline, Patrick G. Cunningham

**Affiliations:** 1 Department of Forest Ecosystems & Society, Oregon State University, Corvallis, OR 97331, USA; 2 Pacific Northwest Research Station, USDA Forest Service, Corvallis, OR 97331, USA

**Keywords:** Projection and prediction, Climate-change impacts, Climate and Earth system modelling

## Abstract

Statistically downscaled climate data have been widely used to explore possible impacts of climate change in various fields of study. Although many studies have focused on characterizing differences in the downscaling methods, few studies have evaluated actual downscaled datasets being distributed publicly. Spatially focusing on the Pacific Northwest, we compare five statistically downscaled climate datasets distributed publicly in the US: ClimateNA, NASA NEX-DCP30, MACAv2-METDATA, MACAv2-LIVNEH and WorldClim. We compare the downscaled projections of climate change, and the associated observational data used as training data for downscaling. We map and quantify the variability among the datasets and characterize the spatio-temporal patterns of agreement and disagreement among the datasets. Pair-wise comparisons of datasets identify the coast and high-elevation areas as areas of disagreement for temperature. For precipitation, high-elevation areas, rainshadows and the dry, eastern portion of the study area have high dissimilarity among the datasets. By spatially aggregating the variability measures into watersheds, we develop guidance for selecting datasets within the Pacific Northwest climate change impact studies.

## Introduction

Projections of historical and future climate from general circulation models (GCM) have been widely used in studies that explore climate change impacts, such as ecology, biogeography, conservation planning, and natural resource management^1–3^. However, GCM outputs are at coarse spatial resolutions (~1 to 3 degrees), and are not sufficiently fine to capture spatial variations of climate in physiographically complex landscapes^4^. Many climate impacts appear only at local scales^5^, and local climate trends do not always follow global trends^6,7^. Consequently, the original GCM outputs may have significant biases in representing the statistical characteristics (e.g., mean, variance, and trend) of local climate conditions^8^. Therefore, the original GCM outputs cannot be directly applied to many fine-scale studies of climate change impacts^9^.

To overcome the inherent limitations of GCM outputs for fine-scale applications, climatologists have developed various techniques to generate fine-resolution climate projections from the coarse-resolution climate change projections output by GCMs^10,11^. The downscaling techniques aim to account for locally or regionally relevant factors that affect climate, such as orographic and coastal effects. Compared with the original GCM outputs, the downscaled climate projections are expected to better represent fine-scale weather patterns, especially for areas with heterogeneous landscape features^12^. In one class of downscaling methods called statistical downscaling, observational climate data from the recent past are used as “training data” to establish statistical relationships between coarse-resolution GCM output and the fine-resolution training data, and those relationships are used to generate fine-resolution future projections from coarse-resolution GCM projections of future climate. Statistical downscaling is relatively computationally efficient, and many statistically downscaled climate change projections have been published online. The published downscaled climate projections provide scientists and land managers an opportunity to explore climate change impacts at local and regional scales, and, in recent years, many statistically downscaled climate datasets have been used to assess the impact of climate change on hydrology^13,14^, species distributions^15^, biodiversity^16^, biological application^17^, and fire modeling^18^.

To date, several studies have compared statistical downscaling methodologies^5,19–21^, such as Constructed Analogues^10^, Bias Corrected Spatial Disaggregation^22^, and Bias Corrected Constructed Analog^23^. Because the primary focus of these comparison studies is the downscaling methodology itself, not the resulting downscaled datasets, the comparison studies use the same GCM output, the same training data, and spatial and temporal resolution and extents to eliminate them as possible sources of differences in the final downscaled data. While this is appropriate for studying the downscaling techniques, the studies do not provide direct assessments of the actual downscaled datasets that are being distributed to the research community and the public. The published datasets may exhibit differences arising from individual GCM bias, the characteristics of the training data, the specific implementation of downscaling algorithms, and data storage and representation issues. To our knowledge only one study, Wootten *et al.*^24^, has compared an ensemble of downscaled datasets that are being distributed to the public. Wootten *et al.*^24^ focuses on data downscaled for the Southeastern USA, and is limited to data downscaled from only one GCM. A similar study is needed for other regions, as well as for other GCMs and downscaling methods. A comprehensive evaluation of downscaled climate datasets can provide greater confidence in the projections of climate change^25^, and, in turn, provide greater confidence in the results of climate change impact studies that rely on them.

In this paper, we compare four downscaled climate datasets published online that cover the Pacific Northwest of USA: ClimateNA (https://adaptwest.databasin.org/pages/adaptwest-climatena/), NASA NEX-DCP30 (https://cds.nccs.nasa.gov/nex/), MACAv2-METDATA (https://climate.northwestknowledge.net/MACA/) and MACAv2-LIVNEH (https://climate.northwestknowledge.net/MACA/). We compare downscaled climate projections generated from six GCMs simulating the Representative Concentration Pathway (RCP) 8.5 climate change scenario. We identify areas of agreement and disagreement, quantify variation across the ensemble of projections, and assess the contribution of the respective training datasets to the differences. Additionally, we compare three of the datasets to WorldClim (http://www.worldclim.org/), another highly cited downscaled climate dataset for which only period averages are published, with no training data. We propose that characterizing the differences among the downscaled datasets provides data users some context for selecting a downscaled climate dataset for a particular application. Finally, we provide summarized data by watersheds at different scales, to serve as guidance for selecting one or more downscaled climate datasets.

## Results

### Comparison of downscaled historical climate

**Figure 1:**
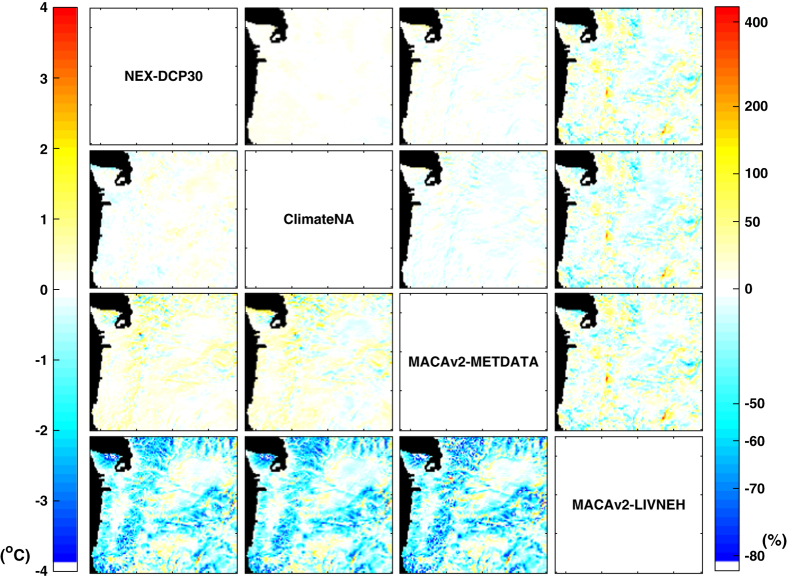
Pair-wise comparison of downscaled climate data for 1961–1990. Dataset names are given along the diagonal. The panels below the diagonal represent differences in mean air temperature for a reference historical period 1961–1990 for every possible pair of downscaled climate datasets. For each panel below the diagonal, the dataset named along the row is subtracted from the dataset named along the column. The panels above the diagonal represent relative differences in mean annual precipitation for 1961–1990. For each panel above the diagonal, the dataset named along the row is divided by the dataset named along the column and 1 is subtracted from the quotient. Note the geometric scale used for precipitation.

Pair-wise comparisons of mean annual temperature and mean annual precipitation from 1961 to 1990 computed from the four downscaled climate datasets show differences among the downscaled datasets, as well as spatial patterns of those differences (Fig. 1). The differences are averaged across the six GCMs common to the four datasets. MACAv2-LIVNEH is the most dissimilar among the compared datasets, in terms of both temperature and precipitation. The mean annual temperature given by MACAv2-LIVNEH is 2-4° C cooler than that given by the other datasets across much of the study area, with the highest differences occurring over mountain ranges and peaks. In terms of mean annual precipitation, MACAv2-LIVNEH deviates from the other datasets across the study area, with values 50–80% higher on the windward slopes of the Cascades, and values 40–50% lower on the leeward slopes of the Cascades, as well as in the interior mountain ranges. These differences can be over 1,000 mm yr^−1^ in absolute precipitation amounts (Supplementary Fig. S1). MACAv2-METDATA exhibits less dissimilarity to NEX-DCP30 and ClimateNA than MACAv2-LIVNEH. Its mean annual temperature values are ~1° C higher than NEX-DCP30 and ClimateNA at all higher elevation portions of the study area; while its precipitation values are lower than those given by NEX-DCP30 and ClimateNA in the Cascades and to the west.

Compared with WorldClim for the period 1950-2000, NEX-DCP30 and MACAv2-METDATA are warmer and wetter, and MACAv2-LIVNEH is cooler and wetter (Table 1). The differences in temperature were due largely to differences in the monthly means of daily minimum temperature (T_min_), where the T_min_ for NEX-DCP30 and MACAv2-METDATA were ~1° C warmer than WorldClim throughout the year, whereas the T_min_ for MACAv2-LIVNEH was ~0.5° C lower than WorldClim. Monthly means of daily maximum temperature (T_max_) of the downscaled datasets deviated from WorldClim by as much as 0.5° C in winter and spring, but it was nearly identical to WorldClim for summer and autumn. All three downscaled datasets compared against WorldClim had higher mean annual precipitation, exceeding WorldClim by 21 to 60 mm per season in the spring, fall and winter.

### Comparison of downscaled future climate projections

**Figure 2:**
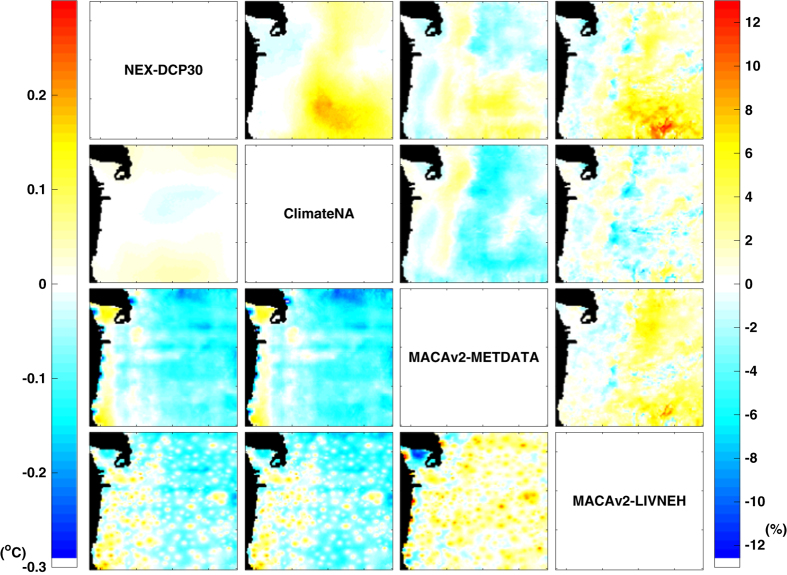
Pair-wise comparison of projected changes temperature and precipitation. The panels below the diagonal represent differences in projected increases in mean air temperature between 1961–1990 and 2071–2100 under RPC8.5 for every possible pair of downscaled climate datasets. The panels above the diagonal represent relative differences in projected changes in mean annual precipitation.

The magnitudes of change in temperature and precipitation projected by the datasets are generally similar to each other (Fig. 2). They vary by only +/− 0.3° C for projected increases in mean annual temperature from 1961-1990 to 2071–2100, which is less than a tenth of the overall climate warming projected for the region by the majority of GCMs. For example, MACAv2-METDATA’s projected warming is only about 0.1° C lower than those projected by NEX-DCP30 and ClimateNA for the eastern half of the study area. Projected magnitudes of change in precipitation generally vary less than 6% among the datasets for most of the study area, with isolated areas of higher differences occurring on the leeward slopes of the Cascades and in the dry, southeastern interior portions of the study area. Dissimilarities between all pairs of datasets are comparable in magnitude and spatial extent, although distinct spatial patterns of differences are displayed. For example, the dissimilarity between NEX-DCP30 and ClimateNA are clustered on the arid southeast portion of the study area, and the difference maps involving MACAv2-LIVNEH exhibit greater spatial heterogeneity. These differences in precipitation are minimized significantly when considered in terms of absolute amount of precipitation (Supplementary Fig. S2). Compared that way, areas of greatest differences in precipitation are generally confined to the windward side of the Cascades.

**Figure 3:**
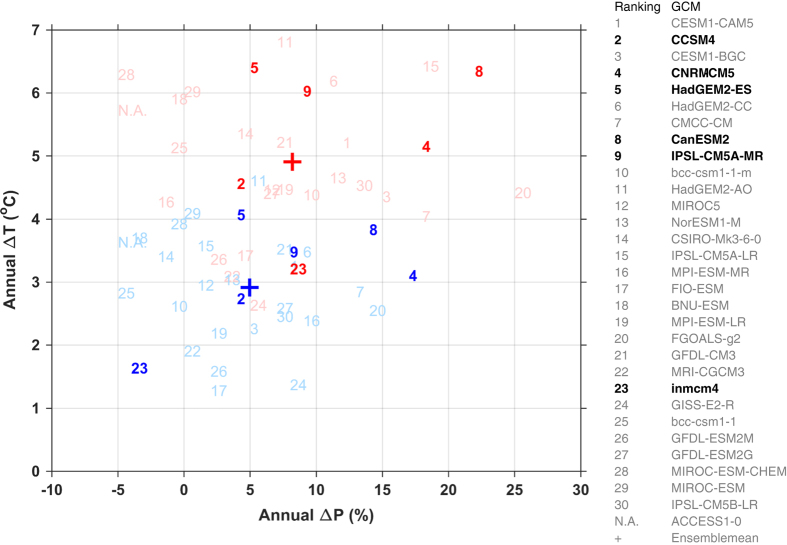
Projections of Climate Change by CMIP5 GCMs with performance ranking. ΔP and ΔT represent changes in mean annual precipitation and mean annual temperature from 1961–1990 to 2071–2100. Blue numbers represent RCP4.5 and red numbers represent RCP8.5, where bold numbers represent GCMs selected in this study. The model performance ranking is from Rupp et al.^43^.

Pair-wise comparisons of downscaled future (2071–2100) climate projections (Supplementary Fig. S3) produce nearly identical results as the pair-wise comparisons of the downscaled historical climate data (Fig. 1) because the projected changes differ relatively little between datasets. As noted in the comparisons of downscaled data for the historical period, projected temperature can differ by as much as 4° C in mountains between two datasets, while projected mean annual precipitation can differ by as much as 2,000 mm yr^−1^ (Supplementary Fig. S3). Again, these are large and significant differences, relative to the 3-7° C warming projected by the ensemble of CMIP5 GCMs under RCP8.5 (Fig. 3) and relative to mean annual precipitation of 974 mm yr^−1^ for the study area.

**Figure 4:**
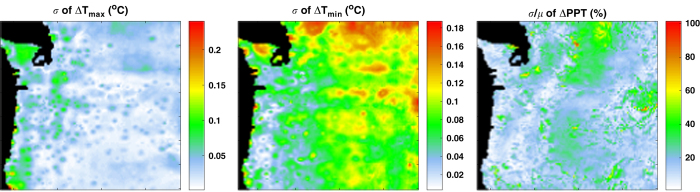
Variability of climate change projections among datasets. Variability in projected magnitudes of change in monthly means of daily maximum and minimum air temperature (∆T_max_ and ∆T_min_, respectively) are shown as standard deviation (*σ*) among the downscaled climate datasets, and variability in projected magnitudes of change in monthly precipitation (∆PPT) is shown as coefficient of variation (*σ/μ*) among the downscaled climate datasets.

Although the projected climates are dissimilar among the downscaled datasets, the magnitudes of climate change projected by the downscaled datasets are highly similar. In other words, even where two datasets project different mean annual temperatures for the 2071-2100 period, the amount of projected warming relative to the reference historical period (1961-1990) are similar. Standard deviations of projected magnitudes of change in annual means of T_max_ and T_min_ from a reference historical period (1961–1990) to the end of the century (2071–2100) is less than 0.25° C for the ensemble of four downscaled climate datasets (Fig. 4). Of the two temperature metrics, there was less agreement among the datasets for T_min_. For projected magnitudes of change in T_max_, the standard deviation of the ensemble was less than 0.05° C for the vast majority of the study area. For projected magnitudes of change in T_min_, the standard deviation was significantly higher in the Cascade Range to the east, with the highest disagreement in northern Washington, where the northern portions of the Cascades and the Northern Rockies are located.

For projected magnitudes of change in mean annual precipitation, the coefficient of variation across the downscaled datasets is generally low west of the Cascade Mountain Range where the precipitation is high. To the east Cascade crest where precipitation is generally lower, however, there are areas where the coefficient of variation ranged 30–60%, representing high levels of disagreement among the downscaled datasets. These areas are located on the leeward side of the Cascade Range and the Wallowa Mountains in Eastern Oregon, where rainshadows occur.

**Figure 5:**
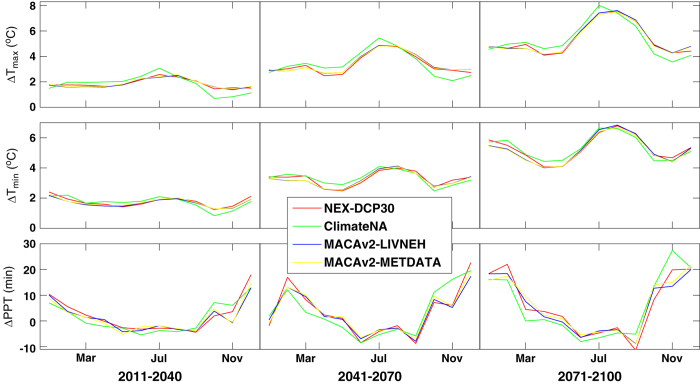
Seasonal patterns of projected climate change. Downscaled projections of change in monthly mean of daily maximum temperature (∆T_max_), monthly mean of daily minimum temperature (∆T_min_), and mean monthly precipitation (∆PPT) under RCP8.5 are plotted for 12 months of the year for the early century (2011–2040), mid-century (2041–2070) and end-century (1971–2100). Change is calculated by averaging across all six common GCMs, relative to the period of 1961–1990.

The differences among the dataset in their projections of change in climate, with respect to the reference historical period (1961–1990), exhibit distinct seasonal patterns that vary throughout the century (Fig. 5). With all four of the downscaled datasets the seasonal cycles become increasingly accentuated from the early- (2011–2040) to mid- (2041–2070) to the end-century (2071–2100): T_max_ becomes increasingly warmer in late summer (July-August); T_min_ increases similarly in both winter and summer; and mean monthly precipitation increases in the winter while decreasing in the summer. In all three time periods considered, ClimateNA’s projections show greater amplitude in seasonal variations compared to the other datasets. Its projections for change in T_max_ are generally greater March to July, while its projections for change in T_min_ are smaller for October and November, compared to the other datasets. ClimateNA also projects a significantly higher increase in November precipitation compared to the other datasets, while projecting smaller increases in winter and spring. These results represent projections of change averaged across six GCMs common to the four datasets. Projected changes by season by individual GCM are detailed in Supplementary Table S1.

**Figure 6:**
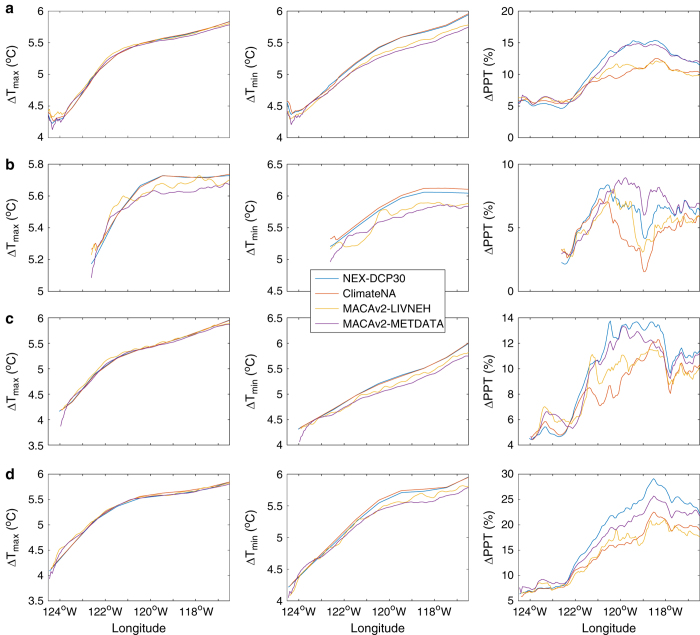
Transect of latitudinally averaged climate variables across the PNW region and three selected transects. Projected magnitudes of change in annual mean of daily maximum temperature (∆T_max_), annual mean of daily minimum temperature (∆T_min_), and the proportional change in mean annual precipitation (∆PPT) from the reference historical period (1961-1990) to the end of the century (2071-2100) per all six common GCMs under RCP8.5 are averaged (**a**) for the study area; (**b**) for a northern transect along the northern border of Washington (48.4^o^N – 49^o^N); (**c**) for a central transect spanning the northern half of Oregon (44.5^o^N – 45.5^o^N); and (**d**) for a southern transect at the southern border of Oregon (42^o^N - 43^o^N).

Projected magnitudes of change in climate from the reference historical period (1961–1990) to the end of the century (2071–2100) vary greatly according to proximity to the coast and by elevation (Fig. 6). Projected magnitudes of change in T_max_ averaged across all latitudes of the study area show a high level of agreement among the four downscaled climate datasets, except at the coast (Fig. 6a). Projected magnitudes of change in T_max_ also disagree at the coast but also diverge significantly east of the Cascades. In terms of projected magnitudes of change in mean annual precipitation, NEX-DCP30 and MACAv2-METDATA project 2–5% greater increase than ClimateNA and MACAv2-LIVNEH in the eastern half of the study area.

Latitudinally averaged transects illustrate the differences among the datasets in representing orographic effects in greater detail. Along the northern transect (48.4–49 °N) (Fig. 6b), which spans the northern Cascades as well as the Rockies, MACAv2-LIVNEH exhibits greater variability in temperature increases over the complex, mountainous topography. NEX-DCP30 and ClimateNA project ~2% greater increases in mean annual precipitation at the crest of the Cascades, with intensifying rainshadow effects immediately east of the crest. Along the central transect (44.5–45.5 °N) (Fig. 6c), MACAv2-METDATA exhibits intensification of the marine effect on temperature, with its projected increase in T_max_ and T_min_ 0.3° C lower than the other three datasets. In terms of precipitation, NEX-DCP30 and MACAv2-METDATA project clearly larger increase in mean annual precipitation over the range of −121.0^o^–−119.0^o^. Along the southern transect (42^o^N–43^o^N) (Fig. 6d), variability in projected increases in T_max_ is notably muted. NEX-DCP30 and ClimateNA show slightly higher increase in T_min_ than the other two datasets over the eastern half of the study area. On the east side of Cascades and further inland, NEX-DCP30 and MACAv2-METDATA project greater increases in precipitation than the other two datasets.

### Differences in training data used for downscaling

**Figure 7:**
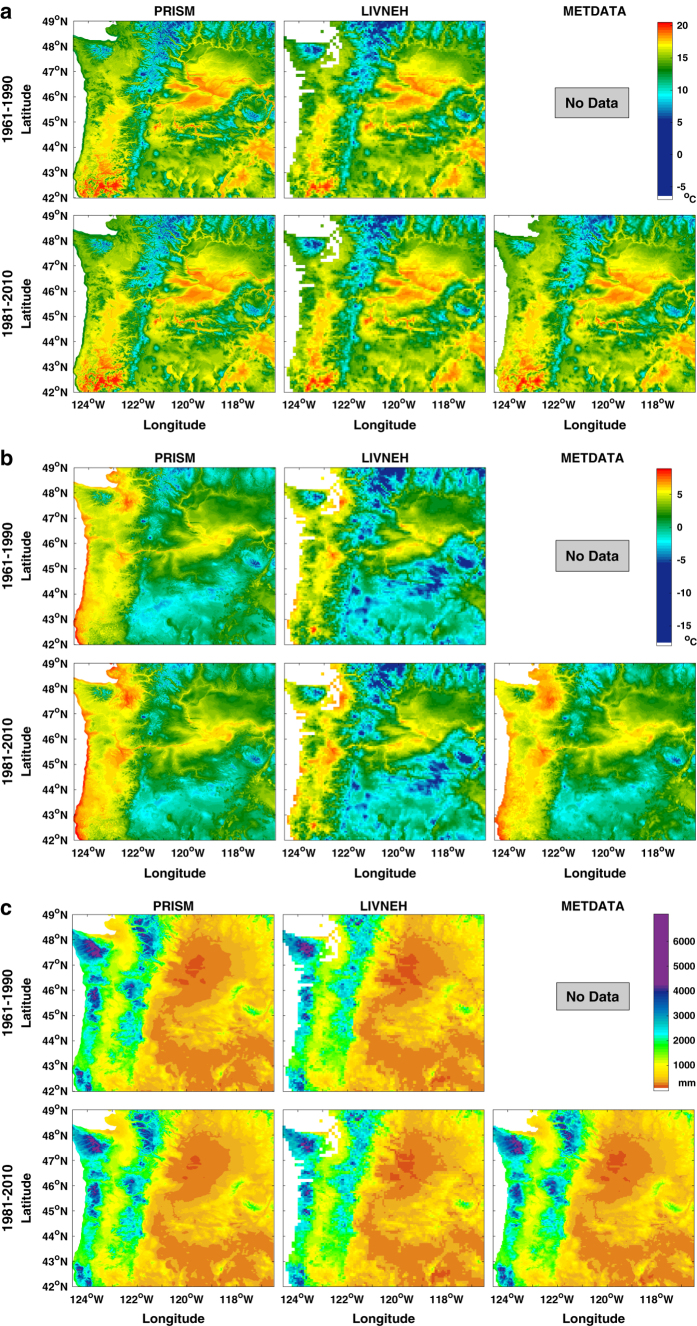
Training data climatology for 1961–1990 and 1981–2010. (**a**) annual mean of daily maximum temperature; (**b**) annual mean of daily minimum temperature; (**c**) annual precipitation.

The observational data shows clear difference among datasets (Table 2). NEX-DCP30 and ClimateNA both use PRISM as the training data. The major difference among training datasets is that LIVNEH has significantly cooler winters, thereby contributing to cooler annual temperatures. In particular, for the period of 1961–1990, LIVNEH has 0.21 °C lower annual T_max_, and 1.39 °C lower T_min_ than PRISM. For period 1981–2010, LIVNEH has 0.17 °C lower annual T_max_, 1.49 °C lower T_min_ than PRISM. In terms of mean annual precipitation and seasonal precipitation, PRISM, METDATA and LIVNEH are similar, each varying from the ensemble average by less than 3%. The three training datasets have similar spatial patterns across the Pacific Northwest region (Fig. 7). The visible difference in T_max_ occurs in Northern Cascades where LIVNEH has lower T_max_ than the other datasets. PRISM and METDATA have similar annual T_min_ in coastal areas, larger than that in LIVNEH. LIVNEH has substantially lower T_min_ than other datasets in the northern Cascades and in the John Day watershed. LIVNEH also shows substantially lower precipitation in the Cascades than the other datasets.

## Discussion

### Guidance for dataset selection

**Figure 8:**
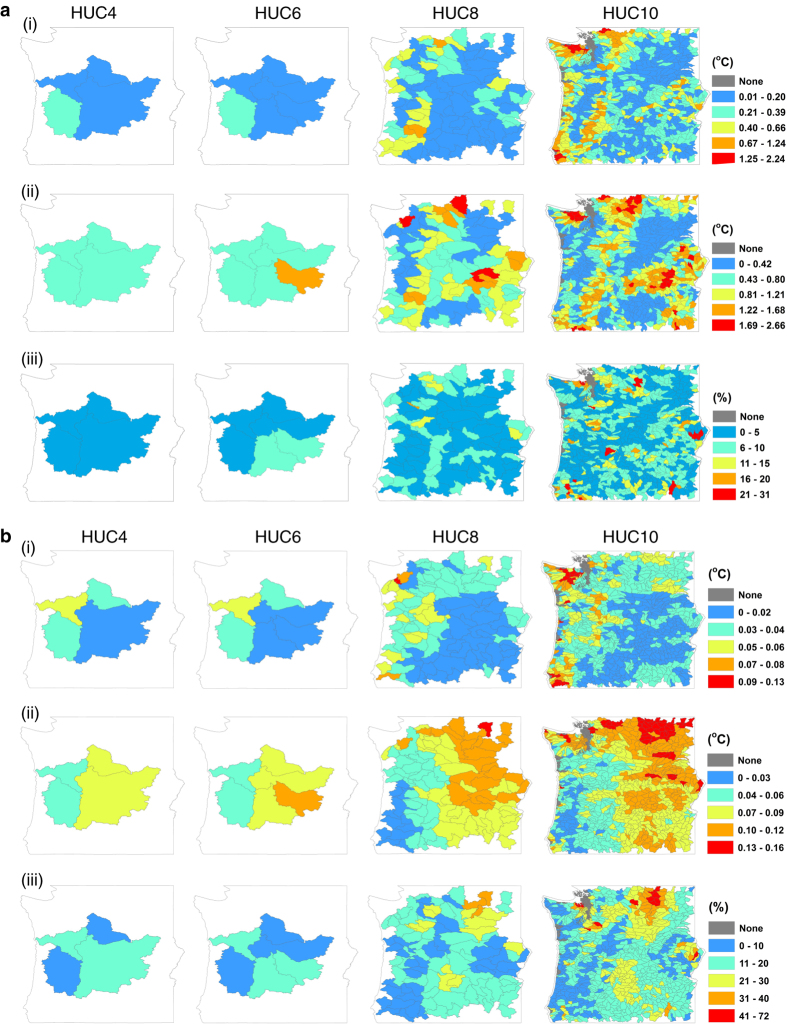
Spatially aggregated variability in projected climate change. (**a**) standard deviation in projected annual means of daily maximum and minimum temperatures, and the coefficient of variation in mean annual precipitation for the period of 2071-2100 under RCP8.5 across the four downscaled climate datasets are spatially averaged to four different levels of US Geological Survey watershed scales (HUC4, HUC6, HUC8 and HUC10)^26^. (**b**) the standard deviation in *change* in annual means of daily maximum and minimum temperatures, and the coefficient of variation in *change* in mean annual precipitation across the four downscaled climate datasets are spatially averaged. Change in temperature and precipitation is calculated for 2071–2100 relative to 1961–1990 reference period.

Variability in projected future (2071–2100) climate among the datasets is spatially aggregated to watersheds to summarize uncertainty of climate change projections across datasets and to aid their selection, application and interpretation (Fig. 8a). Four different scales of watersheds defined by the US Geological Survey^26^ are used. For all three climate variables the differences among datasets are minimized when aggregated to large, regional watersheds. Conversely the differences become more acute for small watersheds. For example, at the US Geological Survey hydrologic unit code level 4 (HUC4) and HUC6 levels^26^, the projected T_max_ of all the watersheds differ by less than 0.4 °C. At the HUC10 level, some watersheds have projections that vary by as much as 2.24 °C. More watersheds are divergent on projections of T_min_ than the other two climate metrics. At the HUC10 level, projections of T_max_ are divergent along the coast and on the western slope of the Cascades, with some watershed having datasets that vary as much as 2.2 °C. Projections of T_min_ are the most divergent in high-elevation watersheds across the study area. With projections of precipitation, several watersheds – but not all – in rainshadows of mountains are indicated as having highly divergent data. Detailed rankings of watersheds at the HUC8 level are given in Supplementary Table S2.

Variability in projected magnitudes of change to climate aggregated to watersheds may aid studies where the magnitudes of change with respect to a reference period, rather than absolute projections of climate, are more relevant (Fig. 8b). As with projected climate aggregated to watersheds, differences among the datasets are minimized when aggregated to large, regional watersheds. For example, differences in changes to T_max_ from 1961–1990 to 2071–2100 among the four downscaled datasets are less than 0.06 °C at HUC4 and HUC6 levels^26^. As with projected climate, projected change in T_min_ is the most divergent, with the vast majority of HUC10 watersheds on the east side of the cascades having relatively more divergent projections of change. For projected magnitudes of change in T_max_, the watersheds with the most divergent datasets are on the west side of the Cascades, especially in the Olympic Peninsula in the northwest corner of the study area. However, as noted previously, the differences in projected magnitudes of change to temperature are small (<0.16 °C). For projected magnitudes of change in precipitation, the drier watersheds in the rainshadows of the Cascades have the most divergent datasets, while the wetter watersheds on the west side of the Cascades exhibit better agreement.

### General discussion

While the differences among the downscaled datasets in their projections of *change* in climate are generally small for much of the study (Fig. 8b), the projections of future climates vary widely for many HUC8 and HUC10 level watersheds the study area (Fig. 8a). These differences may be critical where biological or physical responses to climate change are non-linear or depend on thresholds. For example, in Western Cascades, as mean annual temperature increases, there is an increased rate of reduction in vegetation carbon due to fire activity^27^. In that case, selecting a downscaled dataset that is 2 °C warmer than another has larger consequences than may be inferred from the 2 °C difference. In another example, MC1 dynamic global vegetation model triggers fire suppression efforts based on fireline intensity and rate of spread thresholds^28^. The use of a “warmer” downscaled climate dataset with MC1 instead of a “less warm” dataset would alter whether and when those thresholds are exceeded.

The variability of datasets aggregated by watersheds (Fig. 8) can serve as a practical guide for selecting downscaled datasets for sub-regional-scale studies. Where the variable(s) of interest (i.e., T_max_, T_min_ or precipitation) have a high degree of variability among datasets, there is a greater need to use multiple downscaled datasets to capture the range of future climate projections and the ecological response to those projections. For example, in the Olympic Peninsula in the northwest corner of the study area, there is a relatively high degree of variability among the downscaled datasets, whether their direct climate projections are considered or the magnitude of changes is considered. The average T_max_ and T_min_ may vary by over 2 °C depending on the choice of downscaled climate dataset, while precipitation may vary by over 20%. Using only one downscaled dataset there may severely bias projections of climate change impacts. Conversely, the watershed maps identify areas where there are good agreements among datasets – this is an important benefit, as dataset selection is typically constrained by many criteria: the need for relevant climate variables, the need to seek the finest resolution data (e.g.,<4 km for plant species distributions^29^), maximize accuracy^24^, select high-performing GCMs among the 34 GCMs published by CMIP5, and select appropriate RCP climate change scenarios^30^. Because not every dataset (Table 3) has all the desired qualities, many study areas may be limited to one downscaled dataset. When only one dataset is suitable, yet there is high uncertainty in a study area due to variability among the downscaled datasets, using a large number of GCMs within the single dataset may help address the range of variability across datasets.

Dataset variability maps (Fig. 8) must be interpreted with some caution because large watersheds, e.g., HUC4 watersheds, include the smaller scale watersheds that have high variability of datasets. Therefore, even regional studies must remain cautious about uncertainty of projections in the smaller watersheds, as well as geographic and topographic features where divergence among datasets is observed (Fig. 6). The coast and high-elevation areas are subject to dissimilar temperature projections, and mountain peaks and their rainshadows may have divergent projections of precipitation.

Pair-wise comparisons of datasets for both the historical and future climate yield nearly identical results (Figs. 1 and 2), and the variability of projected magnitudes of changes in climate is small among the datasets (Fig. 4). These results suggest that variability among downscaled climate datasets is mainly controlled by differences in the observational climate data used as training data for the downscaling methods. Indeed, MACAv2-LIVNEH, which had the most dissimilar climate projections to the other datasets, uses LIVNEH training data, which is the most dissimilar to the other training data (Table 2). Comparisons of MACAv2-METDATA with MACAv2-LIVNEH, which uses the same method but different training data, further illustrate the important role of training data. Projected changes in temperature, averaged latitudinally, vary significantly between MACAv2-METDATA and MACAv2-LIVNEH (Fig. 6b), as do projected changes in precipitation. ClimateNA assimilates only one PRISM normal period (for 1971–2000) into its training data, and calculates normals for other periods using CRU-TS 3.22 dataset^31^. This is a potential source of differences between the ClimateNA and NEX-DCP30, and MACAv2-METDATA.

Downscaling methods do partly drive dissimilarity of datasets in some places. We identify two general areas where projections by NEX-DCP30 and MACAv2-METDATA diverge: one, east of the Cascades, projected changes T_min_ diverge by more than 0.2 °C (Fig. 6a); and two, at the Cascade crest, projected changes in mean annual precipitation diverge by more than 2% (Fig. 6b). NEX-DCP30 and MACAv2-METDATA use PRISM and METDATA as training data, respectively, and METDATA is based on PRISM. The uncertainty in high-elevation area likely arises from sparse coverage of weather observations, and poorly understood mechanisms of climate change at fine scales.

The downscaled climate datasets we compare use observational climate data as training data, and those observational data have common weather stations. The datasets and the training data are not independent, and pair-wise comparisons are predisposed toward agreement. For the same reason, a truly independent observational dataset does not exist for evaluating downscaled climate datasets. WorldClim (http://www.worldclim.org/) does not serve as an independent observational dataset to compare the downscaled historical climate data because the weather stations from which it draws its data are partly in common with the training data used by the downscaled datasets. A bootstrapping statistical method may effectively address the lack of independent observational dataset for validation.

We compared a limited number of climate variables because they were the only ones common to the four selected downscaled datasets. All except NEX-DCP30 also include variables representing water vapour (e.g., relative or specific humidity) and wind. ClimateNA is available as a software package, which the user can use to downscale future climate projections for a given area, including 27 bioclimate variables. Comparison of additional variables may reveal other important areas of similarity and dissimilarity. We compared climate variables representing monthly averages and totals (temperature and precipitation, respectively). An important disadvantage of monthly data is that they may not sufficiently represent extreme climate events (e.g., drought, heat wave), which can have critical impact on vegetation^32^, such as forest die-off^33^. MACAv2-METDATA has been demonstrated to be modestly better than data downscaled using BCSD for simulating climate change impacts on wildfire dynamics^11^. The coarse temporal resolution of data hinders exploration of ecological processes with fast response to thermal and water stresses. Data downscaled using Multivariate Adaptive Constructed Analogs (MACA), where climate projections are constructed from a library of observed fine-scale weather patterns, are likely to better represent the occurrence of extremes than those that downscale using average climate surfaces.

## Methods

### Study area

We focused on the Pacific Northwest region of the United States, defined as the geographic area from −124.59^o^ to −116.5^o^ longitude, and from 42^o^ to 49^o^ latitude. The study area is 4.4×10^5^ km^2^ in size and spans two states, Washington and Oregon, in the northwest corner of the conterminous U.S. The climate in the region is Mediterranean, with wet but mild winters, and warm and dry summers. The Cascade Range runs north to south, bisecting the study area. West of the Cascades, moist coniferous forests dominate the landscape. East of the Cascades, the significantly drier climate supports only shrublands and grasslands at lower elevations, while forests occupy higher elevations.

### Downscaled climate datasets

We compared four downscaled climate datasets that are distributed publicly and cover the Pacific Northwest at a relatively high resolution, from 0.5′ to 3.75′: ClimateNA (https://adaptwest.databasin.org/pages/adaptwest-climatena/), NASA NEX-DCP30 (https://cds.nccs.nasa.gov/nex/), MACAv2-METDATA (https://climate.northwestknowledge.net/MACA/), and MACAv2-LIVNEH (https://climate.northwestknowledge.net/MACA/). Because the four selected datasets are described in detail elsewhere^34–36^, we provide only a brief overview of each dataset below, and summarize their key characteristic in Table 3. As a set, the four selected downscaled climate datasets represent multiple statistical downscaling methods applied by multiple institutions, using multiple observational data. Bureau of Reclamation distributes a suite of statistically downscaled climate datasets covering the Pacific Northwest^37^, as does NARCCAP^38^, an ensemble of dynamically downscaled climate datasets, but we excluded both datasets from our analysis because they have significantly coarser spatial resolution (7.5′).

Each statistical downscaling method used to create the four downscaled climate datasets requires the use of an observational climate data as training data. ClimateNA and NEX-DCP30 were created using Parameter-elevation Regressions on Independent Slopes Model (PRISM)^39^ as training data; MACAv2-METDATA used METDATA^36^; and MACAv2-LIVNEH used LIVNEH^40^. To understand the importance training data, we obtained each training dataset, and compared their characteristics.

For comparing future climate projections, we selected two representative concentration pathways^30^, RCP4.5 and RCP8.5, to represent the low and high levels of greenhouse gas forcings^41^. We selected downscaled data from the six Coupled Model Intercomparison Project Phase 5 (CMIP5)^42^ GCMs that are common to all of the four downscaled datasets: CanESM2, CCSM4, CNRM-CM5, HadGEM2-ES, INMCM4, and IPSL-CM5A-MR. The twelve climate projections we evaluated (two RCPs x six GCMs) project a wide range of climate change in the Pacific Northwest by the end of the century in terms of mean annual temperature and precipitation (Fig. 3). Under RCP8.5, the projected rise in temperature ranges over 3 °C among the six selected GCMs, while the projections of change in precipitation range from 5 to over 20%. Notably, all projections we evaluated project an increase in precipitation, with the exception of INMCM4 under RCP4.5, which projects a small decrease in mean annual precipitation. The six selected GCMs have been evaluated for their ability to simulate the recent historical climate of the Pacific Northwest^43^, and all except for INMCM4 are among the top twelve best performers out of the 41 GCMs evaluated. INMCM4 was ranked 30 out of 41. For each of the twelve climate projections we selected three climate variables that are common to all four climate datasets: monthly mean of daily maximum temperature (T_max_), monthly mean of daily minimum temperature (T_min_) and monthly precipitation.

### ClimateNA

ClimateNA uses the Delta method^31^, the simplest of the statistical downscaling methods, to downscale GCM output for North America^35,44–46^. Multiple training datasets are used to cover North America, with PRISM^39^ dataset being used for the conterminous U.S. portion of the dataset^46^. PRISM uses physiographic information to interpolate observations from over 10 000 weather stations into 30″ gridded data^39^. We evaluated the 1km ClimateNA dataset published by A Climate Adaptation Conservation Planning Database for Western North America (AdaptWest) (https://adaptwest.databasin.org/pages/adaptwest-climatena/). Although the downscaling method used by ClimateNA is relatively simple, ClimateNA prioritizes accessibility and utility by spanning the whole continent and including 27 bioclimatic variables. It is also distributed as a software package, so that end-users may generate their own data. ClimateNA dataset published by AdaptWest comprise eight high performing CMIP5 GCMs selected to represent clusters of similar GCMs^47^. The published dataset comprises climate normals for two historical periods, 1961-1990 and 1981-2010, and three future time periods under two climate change scenarios^30^, RCP4.5 and RCP8.5: 2020 s (2011–2040), 2050 s (2041–2070), and 2080 s (2071–2100).

### NEX-DCP30

NEX-DCP30 (https://cds.nccs.nasa.gov/nex/) comprises CMIP5 GCM outputs downscaled to 30″ resolution (approximately 800 m) for the conterminous US using the Bias-Correction Spatial Disaggregation (BCSD) method^34^. BCSD is more sophisticated than the Delta method^43^ in that it uses quantile-mapping to bias-correct the lower resolution GCM data to match the distribution of values in the training data before spatially disaggregates the GCM data to match the finer scale spatial patterns of the training data^19,22,48^. NEX-DCP30 used PRISM^39^ as the training dataset. Among the four datasets we compared, NEX-DCP30 comprised the greatest number of CMIP5 climate projections, including 34 GCMs under four different RCPs. The variables downscaled are limited to just three fundamental metrics – T_max,_ T_min_ and precipitation – but the values are given for every month from 1950 to 2099 for each GCM-RCP combination.

### MACAv2-METDATA

MACAv2-METDATA (https://climate.northwestknowledge.net/MACA/) was generated using the Multivariate Adaptive Constructed Analogs (MACA) method^11^, which builds the downscaled climate data time series by matching coarse-scale GCM patterns to a library of fine-scale observed weather patterns, along with a two-stage of bias correction procedure. The MACA method performs better than BCSD in regions of complex terrain^11^. MACAv2-METDATA uses METDATA^36^ as training data, which combines NASA North American Land Data Assimilation System (NLDAS)^26^ with PRISM^39^. METDATA covers the conterminous US at 2.5’ resolution (approximately 4km) from 1979 to present. The downscaled climate dataset comprises twenty CMIP5 GCMs under two emission scenarios, RCP4.5 and RCP8.5. The data covers the conterminous US at 2.5’ resolution. A suite of 9 climate variables, including maximum and minimum temperature and precipitation, are available at a daily and monthly time step from 1950 to 2099.

### MACAv2-LIVNEH

MACAv2-LIVNEH (https://climate.northwestknowledge.net/MACA/) was also generated using the MACA method^11^, but it was generated using LIVNEH observation dataset as training data. LIVNEH is a long-term hydrologically based dataset of land surface fluxes and states for the conterminous US^40^. It is gridded at a spatial resolution of 1/16 degrees (3.75′ or approximately 6 km), based on interpolated daily temperature and precipitation observations from ~20 000 NOAA Cooperative Observer stations. MACAv2-LIVNEH dataset includes the same ensemble of GCMs and RCPs as MACAv2-METDATA, and the data have the same spatial extent and temporal resolution and extent as MACAv2-METDATA; however, MACAv2-LIVNEH offers a more limited number of climate variables (Table 3).

### WorldClim

WorldClim (http://www.worldclim.org/) covers global lands surfaces except for Antarctica at 30 arc-second resolution. WorldClim’s 1km resolution climate surfaces were created by interpolating weather station data from Global Historical Climatology Network (GHCN)^49^, World Meteorological Organization (WMO) Climatological normals (CLINO)^50^, Food and Agriculture Organization of the United Nations Agroclimatic Database (FAOCLIM)^51^, plus several regional databases. WorldClim uses 14,835 sites for maximum and minimum temperatures and 47,554 sites for precipitation, some of which may be used also in PRISM, METDATA and LIVNEH. The technique used to create WorldClim’s climate surfaces is unique: it uses a thin-plate smoothing spline algorithm to fit a second-order spline where latitude, longitude, and elevation are independent variables^52^. WorldClim publishes data only as period averages: 1960–1999 for the recent historical period, and two future periods, 2041–2060 and 2061–2080. Future data are available for RCP2.6, RCP4.5, RCP6.0 and RCP8.5, for 19 GCMs.

### Comparing the downscaled datasets

NEX-DCP30, MACAv2-METDATA, and MACAv2-LIVNEH publish monthly data online, but ClimateNA and WorldClim publish only period averages. The periods published by ClimateNA and WorldClim do not align. Therefore, we chose to focus our comparison on just four datasets – NEX-DCP30, MACAv2-METDATA, MACAv2-LIVNEH, and ClimateNA – by aligning the periods of analysis with those published by ClimateNA. We chose ClimateNA over WorldClim because ClimateNA has a smaller spatial focus (North America vs. global).

We focus on climate variables that are common to the four downscaled climate datasets we focus on: monthly mean of daily maximum temperature (T_max_), monthly mean of daily minimum temperature (T_min_), and total monthly precipitation downscaled from six GCMs under RCP8.5. MACAv2-LIVNEH downscaled dataset and the LIVNEH training dataset has a spatial resolution of 3.75’ (1/16°), the coarsest among the four downscaled datasets. We interpolated all other datasets to 3.75’ (1/16°) to facilitate comparison.

We examine the differences among the downscaled climate datasets by calculating the differences between every possible pair of datasets for mean annual temperature and mean annual precipitation for two periods, 1961–1990 and 2071–2100 (Figs. 1,2). Mean annual temperature is calculated as the average of annual means of daily maximum and minimum temperatures. Differences are calculated per GCM, and then averaged across the six GCMs common to the four datasets. We examine projected magnitudes of change in climate variables by subtracting the average value from the reference period (1961–1990) from future (2011–2040, 2041–2070, and 2071–2100) period averages.

To aid in the selection and application of downscaled climate datasets in our study area, we created maps of dataset variability for each of the three climate variables we compared, to identify areas with highly divergent climate datasets. To calculate dataset variability, we first calculated the average of six climate projections for 2071–2100 within a dataset, one climate projection per GCM. Then, for T_max_ and T_min_, we calculated standard deviation across the four datasets. The resulting standard deviation grids were aggregated into four levels of watersheds – HUC4, HUC6, HUC8 and HUC10 – defined by the US Geological Survey Watershed Boundary Dataset^26^. For precipitation, we calculated coefficient of variation across the four datasets, so that the measure of variance is scaled by the magnitude precipitation at a given location. We repeated the above steps for projected magnitudes of change in temperature and precipitation for the four levels of watersheds, to create a set of maps that represent variability of magnitudes of change.

To characterize the possible role played by the training datasets in engendering differences among the downscaled datasets, we compared the three training datasets used to create the four downscaled datasets. We calculated seasonal and annual averages for each training dataset and then compared it to the average of the ensemble of the training datasets (Table 2).

As an additional analysis, we compare NEX-DCP30, MACAv2-METDATA and MACAv2-LIVNEH against WorldClim^52^ (http://www.worldclim.org/) by comparing their characteristics for the 1950–2000 period (Table 1). As previously noted, ClimateNA is published online as period averages with different time periods, and is excluded from this comparison.

### Code availability

Matlab code used for data analysis may be obtained freely by contacting the authors, with no restrictions to access.

## Additional information

**How to cite this article:** Jiang, Y. et al. Inter-comparison of multiple statistically downscaled climate datasets for the Pacific Northwest, USA. *Sci. Data* 5:180016 doi: 10.1038/sdata.2018.16 (2018).

**Publisher’s note**: Springer Nature remains neutral with regard to jurisdictional claims in published maps and institutional affiliations.

## Supplementary Material

Supplementary Information

## Figures and Tables

**Table 1:** Comparison of downscaled historical climate data (1950-2000) with WorldClim.

		**NEX-DCP30**	**MACAv2-METDATA**	**MACAv2-LIVNEH**
*T*_*max*_(°C)	Spring	0.3	0.6	0.3
	Summer	0.0	0.1	0.1
	Autumn	0.2	0.0	−0.1
	Winter	0.5	0.5	−0.4
	Annual	0.2	0.3	0.0
*T*_*min*_(°C)	Spring	0.8	1.3	−0.3
	Summer	1.0	1.3	−0.3
	Autumn	1.2	1.3	−0.6
	Winter	1.1	1.1	−0.5
	Annual	1.0	1.2	−0.4
*P*(mm)	Spring	35	48	31
	Summer	5	2	2
	Autumn	33	28	21
	Winter	60	25	32
	Annual	133	103	86
Monthly means of daily maximum and minimum temperatures (*T*_*max*_, and *T*_*min*_, respectively) and monthly precipitation (*P*) for spring (MAM), summer (JJA), autumn (SON), and winter (DJF), as well as annual averages and totals were calculated for NEX-DCP30, MACAv2-METDATA and MACAv2-LIVNEH. Corresponding values from WorldClim were subtracted from those monthly and annual values. The values were calculated as the mean of the six common GCMs. ClimateNA data did not span 1950-2000 and was excluded from this analysis.				

**Table 2:** Differences among the training datasets.

		**1961-1990**			**1981-2010**		
		**PRISM**	**METDATA**	**LIVNEH**	**PRISM**	**METDATA**	**LIVNEH**
							
*T*_*max*_(°C)	Spring	−0.09	-	0.10	−0.17	0.10	0.08
	Summer	−0.06	-	0.07	−0.11	−0.02	0.14
	Autumn	0.18	-	−0.17	0.21	−0.12	−0.08
	Winter	0.40	-	−0.40	0.27	0.35	-0.61
	Annual	0.11	-	−0.10	0.05	0.07	−0.12
*T*_*min*_(°C)	Spring	0.49	-	−0.49	0.29	0.48	−0.76
	Summer	0.63	-	−0.63	0.43	0.48	−0.92
	Autumn	0.92	-	−0.92	0.73	0.52	−1.24
	Winter	0.75	-	−0.75	0.55	0.51	−1.07
	Annual	0.70	-	−0.70	0.50	0.50	−0.99
*P*(%)	Spring	2.3%	-	-2.3%	0.9%	0.6%	-1.5%
	Summer	1.6%	-	−1.6%	1.0%	0.3%	−1.3%
	Autumn	1.8%	-	−1.8%	0.3%	1.6%	−2.0%
	Winter	2.0%	-	−2.0%	1.2%	0.7%	−2.0%
	Annual	2.0%	-	−2.0%	0.9%	0.9%	−1.8%
Differences of each training dataset from the ensemble averages are shown. Variables considered are monthly means of daily maximum and minimum temperatures (*T*_*max*_ and *T*_*min*_, respectively) and monthly precipitation (*P*) for spring (MAM), summer (JJA), autumn (SON), and winter (DJF), as well as annual means and totals. Values were calculated for two historical periods, 1961-1990 and 1981-2010. METDATA did not span 1961-1990.							

**Table 3:** Key characteristics of the downscaled climate datasets compared in this study.

**Climate Dataset**	**Spatial Extent & Resolution**	**Temporal Period(s) & Resolution**	**Downscaling Method**	**Training Data**	**GCMs**	**Climate Scenarios**	**Variables**
ClimateNA	North America @ 1 km	1961-1990, 1981-2010, 2011-2040, 2041-2070, 2071-2100 monthly normals	Delta^31^	PRISM^39^	See footnote A	RCP4.5, RCP8.5	*T*_*min*_, *T*_*max*_, *P*, and 33 other variables
NEX-DCP30	CONUS @ 0.5’	1950-2099 @ monthly	Bias Correction and Spatial Disaggregation (BCSD)^48,22,19^	PRISM^39^	See footnote B	RCP2.6, RCP4.5, RCP6, RCP8.5	*T*_*min*_, *T*_*max*_, *P*
MACAv2-METDATA	CONUS @ 2.5’	1950-2099 @ daily & monthly	Multivariate Adaptive Constructed Analogs (MACA)^11^	METDATA^36^	See footnote C	RCP4.5, RCP8.5	*T*_*min*_, *T*_*max*_, *P*, rhs_min_, rhs_max_, *huss*, *rsds*, *uas*, *vas*
MACAv2-LIVNEH	CONUS @ 3.75’	1950-2099 @ daily & monthly	Multivariate Adaptive Constructed Analogs (MACA)^11^	LIVNEH^40^	See footnote C	RCP4.5, RCP8.5	*T*_*min*_, *T*_*max*_, *P*, *huss*, *rsds*, *was*
Note that ClimateNA data are published as period normals. General circulation models (GCM) included in each downscaled dataset are listed in the footnotes A-D. *T*_*min*_, *T*_*mean*_, and *T*_*max*_ are minimum, mean and maximum temperature, respectively. *P* is precipitation. rhs_min_ and rhs_max_ are minimum and maximum relative humidity, respectively. *huss* is specific humidity. *rsds* is surface downward solar radiation. *was* is wind speed, and *uas* and *vas* are eastward and northward components of wind, respectively.							
^a^CCSM4, CNRM-CM5, CanESM2, INMCM4, GFDL-CM3, HadGEM2-ES, IPSL-CM5A-MR, MPI-ESM-LR.							
^b^ACCESS1-0, FGOALS-g2, IPSL-CM5A-LR, BCC-CSM1-1, FIO-ESM, IPSL-CM5A-MR, BCC-CSM1-1-M, GFDL-CM3, IPSL-CM5B-LR, BNU-ESM, GFDL-ESM2G, MIROC-ESM, CanESM2, GFDL-ESM2M, MIROC-ESM-CHEM, CCSM4, GISS-E2-H-CC, MIROC5, CESM1-BGC, GISS-E2-R, MPI-ESM-LR, CESM1-CAM5, GISS-E2-R-CC, MPI-ESM-MR, CMCC-CM, HadGEM2-AO, MRI-CGCM3, CNRM-CM5, HadGEM2-CC, NorESM1-M, CSIRO-MK3-6-0, HadGEM2-ES, EC-EARTH, INMCM4.							
^c^BCC-CSM1-1, BCC-CSM1-1-M, BNU-ESM, CANESM2, CCSM4, CNRM-CM5, CSIRO-MK3-6-0, GFDL-ESM2G, GFDL-ESM2M, HADGEM2-CC365, HADGEM2-ES365, INMCM4, IPSL-CM5A-LR, IPSL-CM5A-MR, IPSL-CM5B-LR, MIROC5, MIROC-ESM, MIROC-ESM-CHEM, MRI-CGCM3, NORESM1-M.							
