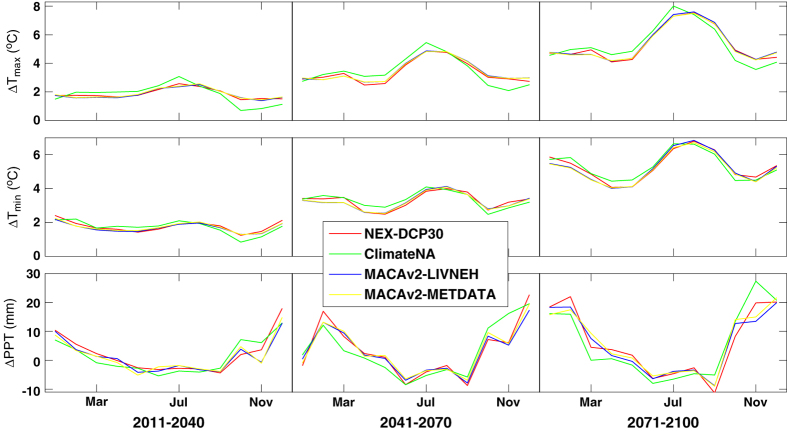# Erratum: Inter-comparison of multiple statistically downscaled climate datasets for the Pacific Northwest, USA

**DOI:** 10.1038/sdata.2018.50

**Published:** 2018-03-27

**Authors:** Yueyang Jiang, John B. Kim, Christopher J. Still, Becky K. Kerns, Jeffrey D. Kline

*Scientific Data* 5:180016 doi: 10.1038/sdata.2018.16 (2018); Published 20 February 2018; Updated 27 March 2018.

In Fig. 5 of this Data Descriptor the bottom-most y-axis label was inadvertently changed from ‘ΔPPT (mm)’ to ‘ΔPPT (min)’ during the production process. The correct version of Fig. 5 appears below as Fig. 1.

## Figures and Tables

**Figure 1:**