# Supplementary material for: Inter-comparison of multiple statistically downscaled climate datasets for the Pacific Northwest, USA
Source: Sci Data. 2018 Feb 20;5:180016. doi: 10.1038/sdata.2018.16 (PMC5819482; doi:10.1038/sdata.2018.16)
Supplement: Supplementary Information [file sdata201816-s1.pdf]

Supplemental Materials

Figure S1. Pair-wise comparison of downscaled climate data for 1961-1990. Dataset names are given along the diagonal. The panels below the diagonal represent differences in mean annual air temperature for a reference historical period 1961-1990 for every possible pair of downscaled climate datasets. For each panel below the diagonal, the dataset named along the row is subtracted from the dataset named along the column. The panels above the diagonal represent differences in mean annual precipitation for 1961-1990. For each panel above the diagonal, the dataset named along the column is subtracted from the dataset named along the row.

Figure S2. Pair-wise comparison of projected changes temperature and precipitation. The panels below the diagonal represent differences in projected increases in mean annual air temperature between 1961-1990 and 2071-2100 under RPC8.5 for every possible pair of downscaled climate datasets. The panels above the diagonal represent differences in projected changes in mean annual precipitation.

Figure S3. Pair-wise comparison of downscaled climate data for end-century. The panels below the diagonal represent differences in mean annual air temperature for end-century (2071-2100) under RPC8.5 for every possible pair of downscaled climate datasets. The panels above the diagonal represent differences in mean annual precipitation for end-century.

Table S1. Projected changes in temperature and precipitation for each of six GCMs.

Table S2. Spatially aggregated variability in projected climate change.

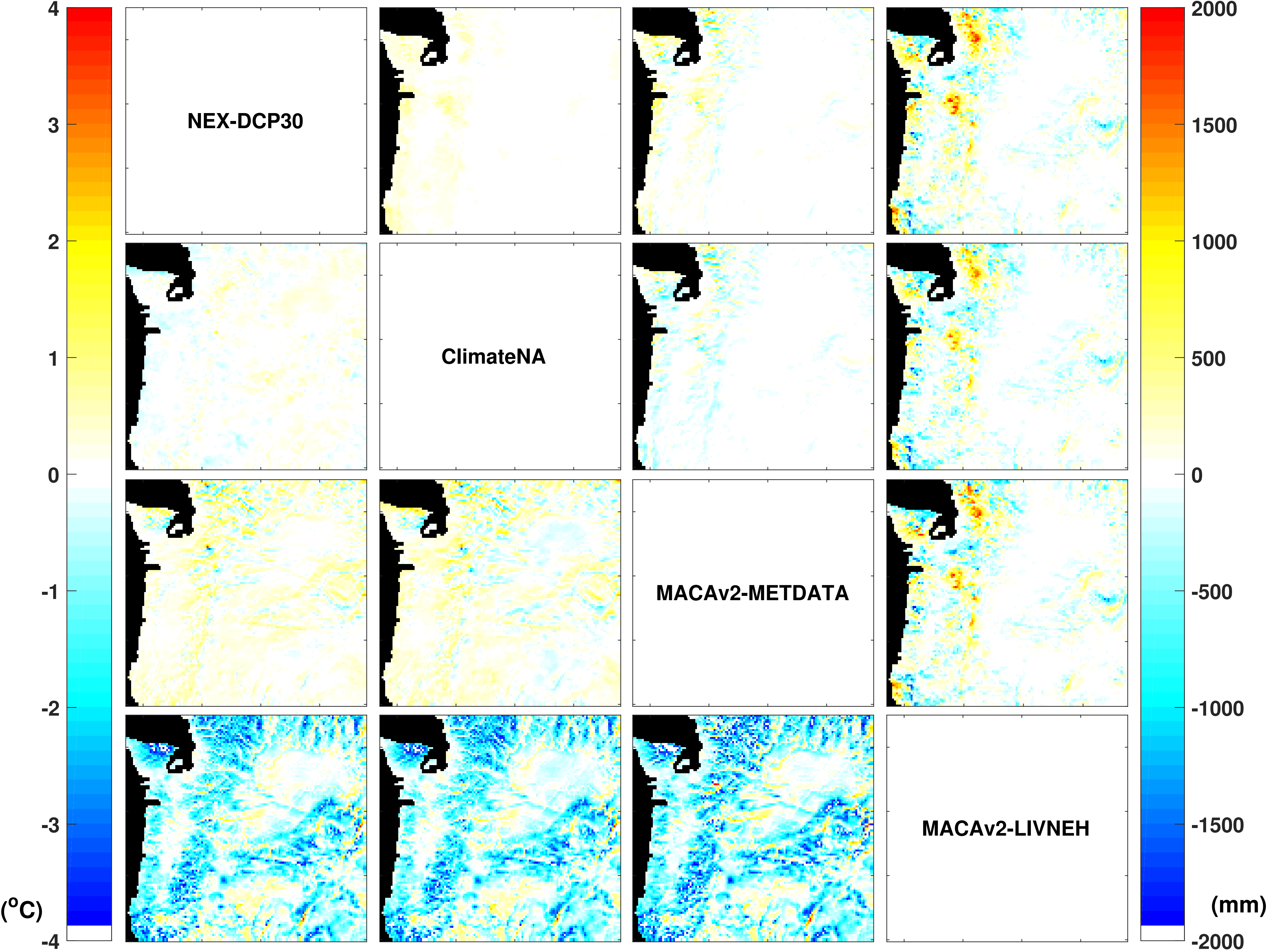

NEX-DCP30
ClimateNA
MACAv2-METDATA
MACAv2-LIVNEH
4
3
2
1
0
-1
-2
-3
-4
(°C)
2000
1500
1000
500
0
-500
-1000
-1500
-2000
(mm)

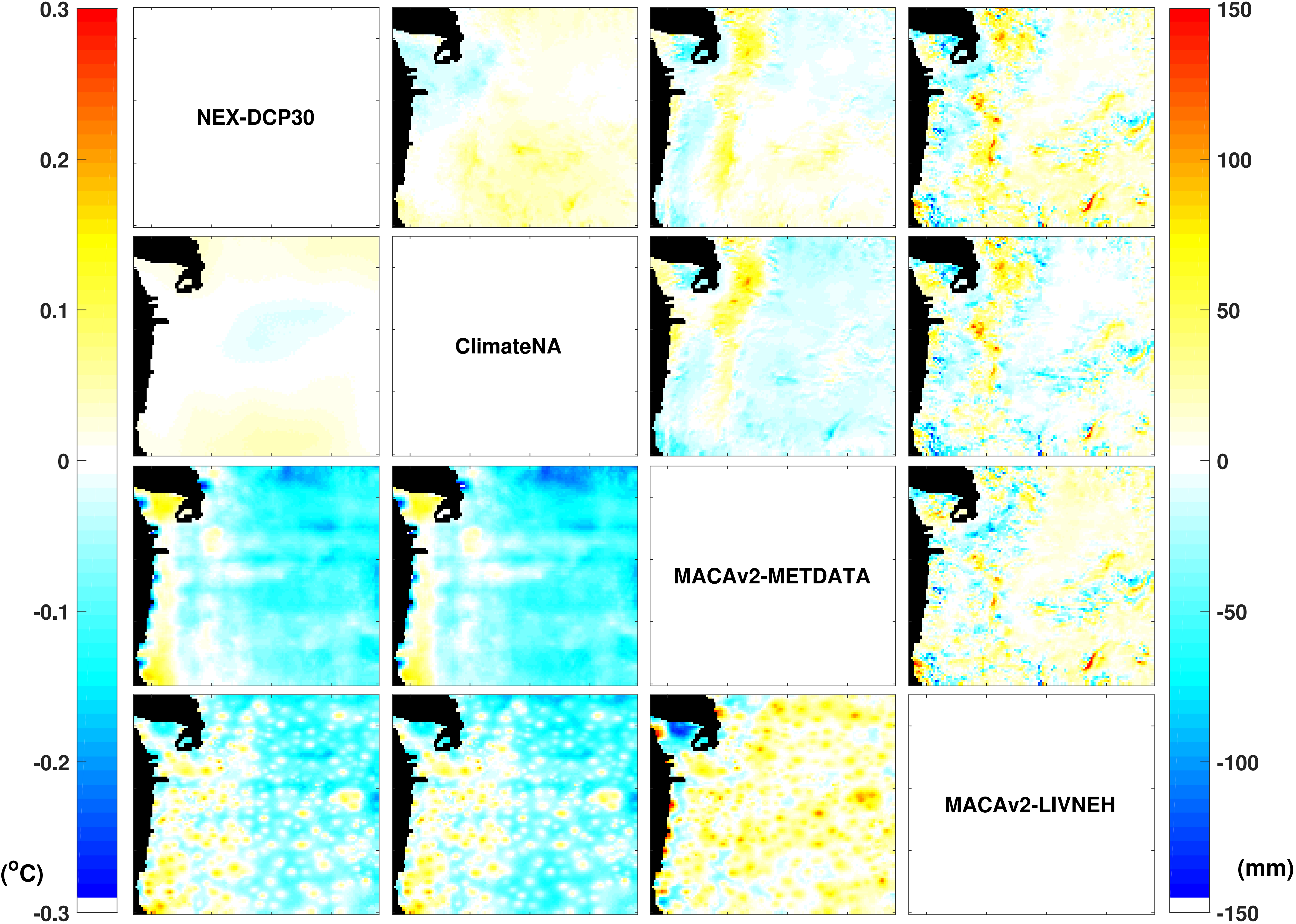
0.3
0.2
0.1
0
-0.1
-0.2
-0.3
(°C)
NEX-DCP30
ClimateNA
MACAv2-METDATA
MACAv2-LIVNEH
150
100
50
0
-50
-100
-150
(mm)

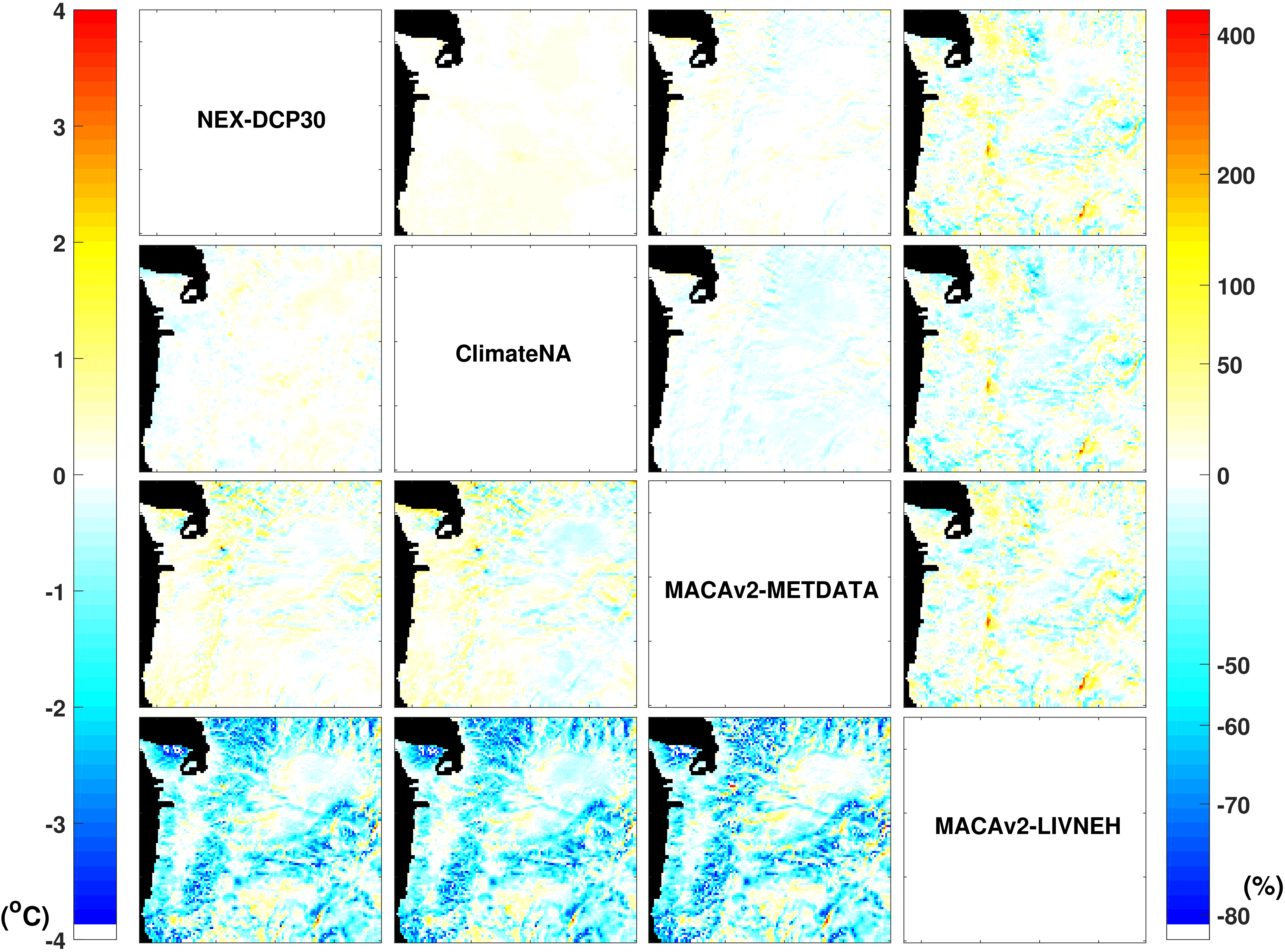
4
3
2
1
0
-1
-2
-3
-4
(°C)
NEX-DCP30
ClimateNA
MACAv2-METDATA
MACAv2-LIVNEH
400
200
100
50
0
-50
-60
-70
-80
(%)

Supplemental Table S1. Projected changes in temperature and precipitation for each of six GCMs. Changes in minimum and maximum temperature and precipitation are summarized by season and annually for climate projections downscaled from individual GCMs under RCP4.5 and RCP8.5. Projected changes are calculated for three future periods (2011-2040, 2041-2070, and 2071-2100) in relation to the 1961-1990 period. (a) Changes in maximum temperature (°C) under RCP4.5 relative to 1961-1990.

Datasets	NEXDCP30	ClimateNA	MACAv2-LIVNEH	MACAv2-METDATA	NEXDCP30	ClimateNA	MACAv2-LIVNEH	MACAv2-METDATA	NEXDCP30	ClimateNA	MACAv2-LIVNEH	MACAv2-METDATA
	2011 - 2040				2041 - 2070				2071 - 2100			
	Spring (Mar-Apr-May)				*Spring (Mar-Apr-May)*				*Spring (Mar-Apr-May)*			
CCSM4	2.07	1.42	1.51	1.49	2.20	2.00	2.50	2.48	2.98	2.45	3.03	2.95
CNRM-CM5	1.91	1.94	1.91	1.97	2.57	2.61	2.61	2.65	2.93	2.95	2.88	2.91
INM-CM4	0.75	0.69	0.80	0.78	0.88	0.79	0.89	0.85	1.21	1.16	1.15	1.18
HadGEM2-ES	1.48	4.29	1.45	1.49	2.58	5.36	2.57	2.61	4.03	6.20	4.07	4.07
CanESM2	1.88	1.88	1.92	1.94	2.26	2.84	2.36	2.35	3.46	3.38	3.44	3.42
IPSL-CM5A-MR	1.47	1.49	1.23	1.27	2.48	2.54	2.46	2.51	2.90	2.91	2.88	2.94
Average	1.59	1.95	1.47	1.49	2.16	2.69	2.23	2.24	2.92	3.18	2.91	2.91
	Summer (Jun-Jul-Aug)				*Summer (Jun-Jul-Aug)*				*Summer (Jun-Jul-Aug)*			
CCSM4	1.88	2.03	2.34	2.33	3.22	3.15	3.44	3.41	3.62	3.52	3.47	3.43
CNRM-CM5	1.55	1.56	1.54	1.54	2.60	2.61	2.64	2.59	3.30	3.33	3.32	3.25
INM-CM4	1.29	1.28	1.32	1.32	2.30	2.34	2.34	2.31	3.38	3.42	3.54	3.40
HadGEM2-ES	2.56	4.36	2.49	2.49	4.49	5.93	4.44	4.39	5.36	6.74	5.41	5.33
CanESM2	3.15	3.11	3.18	3.21	4.71	4.71	4.75	4.78	5.45	5.45	5.47	5.50
IPSL-CM5A-MR	1.87	1.85	1.93	1.93	3.11	3.10	3.29	3.26	3.74	3.74	4.05	3.98
Average	2.05	2.37	2.13	2.14	3.41	3.64	3.48	3.46	4.14	4.37	4.21	4.15
	Autumn (Sep-Oct-Nov)				*Autumn (Sep-Oct-Nov)*				*Autumn (Sep-Oct-Nov)*			
CCSM4	1.80	1.57	1.15	1.13	2.32	2.49	2.04	2.02	2.81	2.72	2.33	2.29
CNRM-CM5	1.15	1.19	1.17	1.16	2.10	2.12	2.12	2.11	2.58	2.59	2.69	2.63
INM-CM4	0.65	0.67	0.71	0.68	1.35	1.34	1.36	1.32	1.88	1.89	1.96	1.89
HadGEM2-ES	1.37	-2.13	1.27	1.28	3.22	-0.56	3.21	3.23	4.19	0.28	4.16	4.12
CanESM2	1.81	1.88	1.77	1.79	2.72	2.82	2.75	2.73	3.11	3.49	3.14	3.10
IPSL-CM5A-MR	1.66	1.71	1.90	1.90	3.52	3.60	3.38	3.43	3.70	3.73	3.89	3.85
Average	1.40	0.81	1.33	1.32	2.54	1.97	2.48	2.47	3.05	2.45	3.03	2.98
	Winter (Dec-Jan-Feb)				*Winter (Dec-Jan-Feb)*				*Winter (Dec-Jan-Feb)*			
CCSM4	1.65	1.42	0.69	0.73	2.16	2.16	2.10	2.07	2.53	2.47	2.50	2.52
CNRM-CM5	1.63	1.66	1.77	1.70	2.70	2.74	2.82	2.74	3.11	3.16	3.08	3.10
INM-CM4	0.64	0.69	0.66	0.72	1.25	1.30	1.20	1.25	1.30	1.37	1.32	1.32
HadGEM2-ES	1.63	0.85	1.50	1.51	2.64	1.92	2.53	2.55	3.66	2.86	3.71	3.71
CanESM2	1.69	1.65	1.74	1.77	2.53	2.75	2.64	2.60	3.20	3.43	3.37	3.30
IPSL-CM5A-MR	1.55	1.60	1.21	1.24	2.70	2.74	2.40	2.44	2.55	2.61	2.71	2.69
Average	1.47	1.31	1.26	1.28	2.33	2.27	2.28	2.27	2.72	2.65	2.78	2.77
	All Year (Jan-Dec)				*All Year (Jan-Dec)*				*All Year (Jan-Dec)*			
CCSM4	1.85	1.61	1.42	1.42	2.48	2.45	2.52	2.49	2.99	2.79	2.83	2.80
CNRM-CM5	1.56	1.59	1.60	1.59	2.49	2.52	2.55	2.53	2.98	3.01	2.99	2.97
INM-CM4	0.83	0.83	0.87	0.88	1.44	1.44	1.45	1.43	1.94	1.96	1.99	1.95
HadGEM2-ES	1.76	1.84	1.68	1.69	3.24	3.16	3.19	3.19	4.31	4.02	4.34	4.31
CanESM2	2.13	2.13	2.15	2.18	3.06	3.28	3.12	3.12	3.80	3.94	3.86	3.83
IPSL-CM5A-MR	1.64	1.66	1.57	1.59	2.95	2.99	2.88	2.91	3.22	3.25	3.38	3.37
Average	1.63	1.61	1.55	1.56	2.61	2.64	2.62	2.61	3.21	3.16	3.23	3.20

(b) Changes in maximum temperature (^{o}C) under RCP8.5 relative to 1961-1990.

Datasets	NEXDCP30	ClimateNA	MACAv2-LIVNEH	MACAv2-METDATA	NEXDCP30	ClimateNA	MACAv2-LIVNEH	MACAv2-METDATA	NEXDCP30	ClimateNA	MACAv2-LIVNEH	MACAv2-METDATA
	2011 - 2040				2041 - 2070				2071 - 2100			
	Spring (Mar-Apr-May)				*Spring (Mar-Apr-May)*				*Spring (Mar-Apr-May)*			
CCSM4	2.03	1.38	1.93	1.91	2.95	2.62	3.12	3.08	4.24	4.02	3.80	3.74
CNRM-CM5	1.94	1.94	1.91	1.99	2.50	2.53	2.43	2.51	4.66	4.68	4.73	4.70
INM-CM4	0.58	0.56	0.63	0.62	1.40	1.34	1.48	1.45	1.90	1.84	1.82	1.87
HadGEM2-ES	2.02	4.40	1.94	2.01	3.66	6.31	3.68	3.72	5.78	8.42	5.83	5.82
CanESM2	1.92	1.88	1.94	1.95	3.11	3.48	3.23	3.17	5.22	5.28	5.23	5.20
IPSL-CM5A-MR	1.78	1.80	1.62	1.70	3.08	3.14	3.07	3.10	4.82	4.87	4.85	4.95
Average	1.71	1.99	1.66	1.70	2.78	3.24	2.84	2.84	4.44	4.85	4.38	4.38
	Summer (Jun-Jul-Aug)				*Summer (Jun-Jul-Aug)*				*Summer (Jun-Jul-Aug)*			
CCSM4	2.30	2.30	2.37	2.40	3.99	4.12	4.18	4.16	6.62	6.53	6.69	6.54
CNRM-CM5	1.90	1.89	1.82	1.87	3.43	3.43	3.45	3.40	5.77	5.82	5.83	5.70
INM-CM4	2.00	2.04	2.05	2.06	3.90	3.97	4.03	3.96	5.26	5.30	5.35	5.21
HadGEM2-ES	2.85	4.36	2.70	2.75	5.48	7.26	5.45	5.41	8.85	10.49	8.93	8.83
CanESM2	3.19	3.26	3.22	3.26	5.82	6.00	5.80	5.87	8.74	8.97	8.69	8.70
IPSL-CM5A-MR	1.98	1.96	2.07	2.09	4.37	4.37	4.44	4.39	6.29	6.28	6.56	6.46
Average	2.37	2.64	2.37	2.41	4.50	4.86	4.56	4.53	6.92	7.23	7.01	6.91
	Autumn (Sep-Oct-Nov)				*Autumn (Sep-Oct-Nov)*				*Autumn (Sep-Oct-Nov)*			
CCSM4	1.58	1.73	1.54	1.53	3.22	3.36	3.07	3.04	5.16	4.89	4.50	4.48
CNRM-CM5	1.89	1.88	1.84	1.87	2.95	2.97	3.00	2.96	4.74	4.77	4.84	4.72
INM-CM4	1.31	1.34	1.33	1.31	2.02	2.01	2.09	2.04	3.64	3.66	3.72	3.66
HadGEM2-ES	1.91	-1.87	1.85	1.85	4.03	0.36	4.07	4.05	6.11	2.68	6.15	6.10
CanESM2	1.61	1.89	1.60	1.64	3.62	4.01	3.59	3.61	6.15	6.11	6.07	6.06
IPSL-CM5A-MR	1.81	1.83	1.83	1.86	3.96	4.00	4.61	4.57	6.14	6.22	6.72	6.67
Average	1.68	1.13	1.67	1.68	3.30	2.79	3.41	3.38	5.32	4.72	5.34	5.28
	Winter (Dec-Jan-Feb)				*Winter (Dec-Jan-Feb)*				*Winter (Dec-Jan-Feb)*			
CCSM4	1.60	1.65	1.59	1.60	2.23	2.46	2.52	2.52	3.94	4.13	4.45	4.41
CNRM-CM5	1.76	1.79	1.91	1.84	2.87	2.91	2.92	2.87	5.07	5.09	5.10	5.05
INM-CM4	0.87	0.93	0.85	0.91	1.44	1.52	1.48	1.53	2.83	2.93	2.88	2.86
HadGEM2-ES	1.74	1.01	1.52	1.56	4.06	3.18	3.97	3.97	5.90	5.23	5.86	5.86
CanESM2	1.99	1.74	2.01	2.09	3.39	3.51	3.45	3.44	5.00	5.03	5.13	5.07
IPSL-CM5A-MR	2.08	2.07	2.00	2.06	3.34	3.33	3.15	3.23	4.78	4.80	4.96	4.91
Average	1.67	1.53	1.65	1.68	2.89	2.82	2.91	2.93	4.59	4.53	4.73	4.69
	All Year (Jan-Dec)				*All Year (Jan-Dec)*				*All Year (Jan-Dec)*			
CCSM4	1.88	1.76	1.85	1.86	3.10	3.14	3.22	3.20	4.99	4.89	4.86	4.79
CNRM-CM5	1.87	1.88	1.87	1.89	2.94	2.96	2.95	2.94	5.06	5.09	5.13	5.04
INM-CM4	1.19	1.22	1.21	1.23	2.19	2.21	2.27	2.25	3.41	3.43	3.44	3.40
HadGEM2-ES	2.13	1.98	2.00	2.04	4.31	4.28	4.29	4.29	6.66	6.70	6.69	6.65
CanESM2	2.18	2.19	2.19	2.24	3.99	4.25	4.02	4.02	6.28	6.34	6.28	6.26
IPSL-CM5A-MR	1.91	1.91	1.88	1.93	3.69	3.71	3.82	3.82	5.51	5.54	5.77	5.75
Average	1.86	1.82	1.84	1.86	3.37	3.42	3.43	3.42	5.32	5.33	5.36	5.32

(c) Changes in minimum temperature (^{o}C) under RCP4.5 relative to 1961-1990.

Datasets	NEXDCP30	ClimateNA	MACAv2-LIVNEH	MACAv2-METDATA	NEXDCP30	ClimateNA	MACAv2-LIVNEH	MACAv2-METDATA	NEXDCP30	ClimateNA	MACAv2-LIVNEH	MACAv2-METDATA
	2011 - 2040				2041 - 2070				2071 - 2100			
	Spring (Mar-Apr-May)				*Spring (Mar-Apr-May)*				*Spring (Mar-Apr-May)*			
CCSM4	1.32	1.11	0.96	0.95	1.43	1.56	1.58	1.58	2.23	1.99	2.15	2.10
CNRM-CM5	1.91	2.01	2.02	2.05	2.76	2.88	2.86	2.87	3.28	3.40	3.42	3.41
INM-CM4	0.43	0.47	0.55	0.51	0.97	1.06	1.10	1.08	1.37	1.43	1.37	1.41
HadGEM2-ES	1.44	3.00	1.42	1.45	2.29	3.89	2.27	2.31	3.20	4.51	3.26	3.27
CanESM2	1.99	1.92	2.02	2.05	2.60	2.91	2.70	2.69	3.48	3.45	3.55	3.57
IPSL-CM5A-MR	1.36	1.21	1.12	1.11	2.60	2.45	2.11	2.17	2.66	2.56	2.46	2.51
Average	1.41	1.62	1.35	1.35	2.11	2.46	2.10	2.11	2.70	2.89	2.70	2.71
	Summer (Jun-Jul-Aug)				*Summer (Jun-Jul-Aug)*				*Summer (Jun-Jul-Aug)*			
CCSM4	1.51	1.55	1.65	1.65	2.19	2.33	2.44	2.40	2.67	2.64	2.68	2.64
CNRM-CM5	1.55	1.60	1.58	1.60	2.34	2.38	2.40	2.39	3.14	3.21	3.24	3.18
INM-CM4	0.39	0.34	0.41	0.38	0.71	0.64	0.74	0.68	1.18	1.14	1.26	1.10
HadGEM2-ES	1.91	2.80	1.86	1.88	3.53	4.23	3.58	3.55	4.54	5.16	4.64	4.57
CanESM2	2.75	2.61	2.77	2.79	4.28	4.20	4.39	4.36	5.13	5.10	5.29	5.23
IPSL-CM5A-MR	1.74	1.73	1.99	2.02	3.07	3.13	3.14	3.19	3.85	3.87	3.99	4.00
Average	1.64	1.77	1.71	1.72	2.69	2.82	2.78	2.76	3.42	3.52	3.52	3.45
	Autumn (Sep-Oct-Nov)				*Autumn (Sep-Oct-Nov)*				*Autumn (Sep-Oct-Nov)*			
CCSM4	1.27	1.18	0.94	0.97	1.85	2.00	1.98	1.95	2.39	2.24	2.05	2.03
CNRM-CM5	1.33	1.33	1.26	1.29	2.11	2.14	2.16	2.13	2.80	2.82	2.85	2.85
INM-CM4	0.42	0.36	0.42	0.40	0.46	0.41	0.54	0.49	1.09	1.03	1.19	1.13
HadGEM2-ES	1.36	-0.45	1.29	1.27	3.07	0.97	3.16	3.14	4.05	1.72	4.09	4.01
CanESM2	1.79	1.57	1.72	1.74	2.82	2.74	2.78	2.79	3.08	3.38	3.05	3.06
IPSL-CM5A-MR	1.56	1.71	1.89	1.91	2.74	2.98	2.93	3.00	3.75	3.89	3.72	3.72
Average	1.29	0.95	1.25	1.26	2.18	1.87	2.26	2.25	2.86	2.51	2.83	2.80
	Winter (Dec-Jan-Feb)				*Winter (Dec-Jan-Feb)*				*Winter (Dec-Jan-Feb)*			
CCSM4	1.59	1.49	0.73	0.83	2.33	2.34	2.34	2.32	2.61	2.69	2.84	2.89
CNRM-CM5	2.05	2.11	2.13	2.05	3.29	3.34	3.40	3.25	3.83	3.88	3.86	3.81
INM-CM4	1.05	1.12	1.08	1.13	1.81	1.98	1.84	1.91	1.82	1.95	1.87	1.90
HadGEM2-ES	1.62	0.79	1.56	1.58	2.67	1.92	2.63	2.62	3.59	2.84	3.63	3.64
CanESM2	1.96	1.99	2.08	2.07	3.10	3.42	3.29	3.23	3.94	4.24	4.19	4.09
IPSL-CM5A-MR	1.95	1.86	1.33	1.34	3.68	3.63	2.48	2.49	4.45	4.36	2.96	2.95
Average	1.70	1.56	1.48	1.50	2.82	2.77	2.66	2.64	3.38	3.33	3.22	3.21
	All Year (Jan-Dec)				*All Year (Jan-Dec)*				*All Year (Jan-Dec)*			
CCSM4	1.42	1.33	1.07	1.10	1.95	2.06	2.08	2.06	2.47	2.39	2.43	2.41
CNRM-CM5	1.71	1.76	1.75	1.75	2.62	2.69	2.71	2.66	3.26	3.33	3.34	3.31
INM-CM4	0.57	0.57	0.61	0.60	0.99	1.02	1.05	1.04	1.36	1.39	1.42	1.38
HadGEM2-ES	1.58	1.53	1.53	1.55	2.89	2.75	2.91	2.90	3.85	3.56	3.91	3.87
CanESM2	2.12	2.02	2.15	2.16	3.20	3.32	3.29	3.27	3.91	4.04	4.02	3.99
IPSL-CM5A-MR	1.65	1.63	1.58	1.60	3.02	3.05	2.66	2.71	3.68	3.67	3.28	3.30
Average	1.51	1.48	1.45	1.46	2.45	2.48	2.45	2.44	3.09	3.06	3.07	3.04

(d) Changes in minimum temperature (^{o}C) under RCP8.5 relative to 1961-1990.

Datasets	NEXDCP30	ClimateNA	MACAv2-LIVNEH	MACAv2-METDATA	NEXDCP30	ClimateNA	MACAv2-LIVNEH	MACAv2-METDATA	NEXDCP30	ClimateNA	MACAv2-LIVNEH	MACAv2-METDATA
	2011 - 2040				2041 - 2070				2071 - 2100			
	Spring (Mar-Apr-May)				*Spring (Mar-Apr-May)*				*Spring (Mar-Apr-May)*			
CCSM4	1.44	1.13	1.19	1.19	2.22	2.09	2.24	2.21	3.19	3.12	2.76	2.72
CNRM-CM5	2.13	2.24	2.19	2.22	3.10	3.20	3.14	3.19	4.96	5.09	5.15	5.11
INM-CM4	0.66	0.71	0.74	0.75	1.55	1.63	1.68	1.65	2.66	2.74	2.70	2.76
HadGEM2-ES	1.65	3.10	1.58	1.64	3.07	4.66	3.09	3.10	4.93	6.53	5.01	5.00
CanESM2	2.08	1.99	2.12	2.18	3.53	3.57	3.60	3.59	5.27	5.25	5.36	5.34
IPSL-CM5A-MR	1.40	1.13	1.16	1.29	3.64	3.56	2.85	2.87	5.04	4.93	4.32	4.41
Average	1.56	1.71	1.50	1.54	2.85	3.12	2.77	2.77	4.34	4.61	4.22	4.22
	Summer (Jun-Jul-Aug)				*Summer (Jun-Jul-Aug)*				*Summer (Jun-Jul-Aug)*			
CCSM4	1.87	1.77	1.79	1.80	3.12	3.26	3.25	3.22	5.34	5.14	5.16	5.07
CNRM-CM5	1.86	1.91	1.83	1.90	3.25	3.29	3.30	3.27	5.49	5.53	5.61	5.48
INM-CM4	0.47	0.42	0.43	0.42	1.17	1.11	1.28	1.14	2.72	2.67	2.84	2.66
HadGEM2-ES	2.27	2.97	2.18	2.26	4.51	5.48	4.52	4.50	7.83	8.63	8.02	7.90
CanESM2	2.76	2.80	2.78	2.80	5.61	5.55	5.68	5.68	8.72	8.70	8.83	8.73
IPSL-CM5A-MR	1.72	1.75	2.00	2.08	4.02	4.06	4.38	4.43	6.34	6.40	6.64	6.63
Average	1.83	1.94	1.84	1.88	3.61	3.79	3.74	3.71	6.07	6.18	6.18	6.08
	Autumn (Sep-Oct-Nov)				*Autumn (Sep-Oct-Nov)*				*Autumn (Sep-Oct-Nov)*			
CCSM4	1.38	1.48	1.36	1.35	2.61	2.86	2.53	2.52	4.24	4.24	4.20	4.14
CNRM-CM5	1.61	1.60	1.58	1.57	3.00	3.02	3.02	3.02	4.86	4.89	4.91	4.89
INM-CM4	0.52	0.46	0.49	0.48	1.21	1.17	1.30	1.25	2.79	2.78	2.92	2.79
HadGEM2-ES	1.93	-0.29	1.84	1.85	3.96	1.89	3.96	3.91	6.05	4.39	6.21	6.12
CanESM2	1.73	1.80	1.61	1.68	3.60	3.80	3.55	3.57	6.26	6.09	6.25	6.20
IPSL-CM5A-MR	1.81	1.99	1.72	1.76	5.05	5.21	4.46	4.48	7.36	7.62	6.67	6.68
Average	1.50	1.17	1.43	1.45	3.24	2.99	3.14	3.12	5.26	5.00	5.19	5.14
	Winter (Dec-Jan-Feb)				*Winter (Dec-Jan-Feb)*				*Winter (Dec-Jan-Feb)*			
CCSM4	1.70	1.77	1.77	1.84	2.26	2.68	2.95	2.99	4.08	4.40	4.77	4.74
CNRM-CM5	2.23	2.29	2.32	2.23	3.55	3.65	3.66	3.58	6.04	6.09	6.10	6.02
INM-CM4	1.35	1.48	1.38	1.41	1.65	1.80	1.81	1.83	3.96	4.12	4.02	4.05
HadGEM2-ES	1.94	0.95	1.73	1.84	3.94	3.03	3.91	3.88	5.69	4.93	5.79	5.74
CanESM2	2.23	2.19	2.37	2.38	4.00	4.35	4.17	4.11	6.00	6.12	6.18	6.05
IPSL-CM5A-MR	3.44	3.52	2.16	2.19	4.96	4.79	3.28	3.38	7.63	7.62	5.19	5.16
Average	2.15	2.04	1.96	1.98	3.39	3.38	3.30	3.29	5.57	5.55	5.34	5.29
	All Year (Jan-Dec)				*All Year (Jan-Dec)*				*All Year (Jan-Dec)*			
CCSM4	1.60	1.54	1.53	1.54	2.55	2.72	2.74	2.73	4.21	4.22	4.22	4.17
CNRM-CM5	1.96	2.01	1.98	1.98	3.22	3.29	3.28	3.26	5.34	5.40	5.44	5.37
INM-CM4	0.75	0.77	0.76	0.77	1.39	1.43	1.52	1.47	3.03	3.08	3.12	3.07
HadGEM2-ES	1.95	1.68	1.83	1.90	3.87	3.77	3.87	3.85	6.13	6.12	6.26	6.19
CanESM2	2.20	2.19	2.22	2.26	4.18	4.32	4.25	4.24	6.56	6.54	6.65	6.58
IPSL-CM5A-MR	2.10	2.10	1.76	1.83	4.42	4.40	3.75	3.79	6.59	6.64	5.71	5.72
Average	1.76	1.71	1.68	1.71	3.27	3.32	3.23	3.22	5.31	5.33	5.23	5.18

(e) Changes in seasonal and annual precipitation (mm) under RCP4.5 relative to 1961-1990.

Datasets	NEXDCP30	ClimateNA	MACAv2-LIVNEH	MACAv2-METDATA	NEXDCP30	ClimateNA	MACAv2-LIVNEH	MACAv2-METDATA	NEXDCP30	ClimateNA	MACAv2-LIVNEH	MACAv2-METDATA
	2011 - 2040				2041 - 2070				2071 - 2100			
	Spring (Mar-Apr-May)				*Spring (Mar-Apr-May)*				*Spring (Mar-Apr-May)*			
CCSM4	-29.4	-5.6	-22.9	-29.1	-12.2	-0.4	-11.2	-16.0	-5.2	6.3	-3.1	-5.9
CNRM-CM5	4.5	0.9	-3.6	-1.4	13.4	8.7	6.5	8.8	23.6	19.3	19.1	25.5
INM-CM4	-13.1	-10.1	-10.0	-7.7	2.5	5.7	0.4	1.8	-1.8	-1.0	-5.7	-5.4
HadGEM2-ES	20.5	-30.4	17.7	23.8	24.5	-27.9	21.2	26.2	0.9	-33.7	-0.1	2.6
CanESM2	19.8	4.0	18.7	17.8	26.0	5.2	25.3	26.9	21.8	7.7	20.5	20.8
IPSL-CM5A-MR	1.4	0.0	1.8	0.0	-4.2	-2.6	-0.1	-2.1	-3.7	-2.1	-0.5	-4.0
Average	0.6	-6.9	0.3	0.6	8.3	-1.9	7.0	7.6	5.9	-0.6	5.0	5.6
	Summer (Jun-Jul-Aug)				*Summer (Jun-Jul-Aug)*				*Summer (Jun-Jul-Aug)*			
CCSM4	-0.7	-3.1	-7.4	-10.0	-12.0	-5.6	-9.1	-15.4	-8.7	-6.5	2.7	0.2
CNRM-CM5	0.8	-0.8	-1.4	10.0	-13.8	-10.6	-10.9	-3.2	-3.7	-4.5	-2.6	11.2
INM-CM4	-14.3	-9.3	-11.7	-12.3	-27.5	-19.3	-16.8	-19.9	-35.5	-25.5	-23.1	-23.7
HadGEM2-ES	-16.1	-40.3	-17.1	-16.9	-20.5	-41.0	-23.1	-23.6	-17.1	-38.0	-17.8	-19.2
CanESM2	-4.2	-15.3	-9.5	-8.6	3.4	-9.9	-1.7	-7.8	11.7	-1.6	7.2	3.3
IPSL-CM5A-MR	4.1	2.3	3.0	6.8	-15.0	-16.6	-14.7	-14.0	-7.9	-9.9	-8.3	-8.5
Average	-5.1	-11.1	-7.3	-5.2	-14.2	-17.2	-12.7	-14.0	-10.2	-14.3	-7.0	-6.1
	Autumn (Sep-Oct-Nov)				*Autumn (Sep-Oct-Nov)*				*Autumn (Sep-Oct-Nov)*			
CCSM4	-0.4	-3.4	-9.6	-15.1	27.6	-3.6	21.1	22.1	19.7	1.0	15.1	14.6
CNRM-CM5	22.1	27.7	18.1	21.6	10.0	12.6	8.5	12.6	53.6	56.6	46.4	58.1
INM-CM4	-0.5	3.1	3.6	5.1	-20.3	-14.0	-12.5	-6.6	-22.5	-16.5	-18.8	-14.2
HadGEM2-ES	-5.4	97.5	-4.5	-5.1	15.1	107.5	18.4	18.6	28.7	102.6	27.9	34.4
CanESM2	5.2	-1.6	5.1	5.9	-5.8	5.7	-3.6	-7.8	8.0	9.4	9.8	12.6
IPSL-CM5A-MR	-17.1	-15.2	-11.9	-11.8	-34.6	-35.4	-29.8	-27.8	8.7	10.6	12.3	14.5
Average	0.6	18.0	0.1	0.1	-1.4	12.1	0.4	1.9	16.0	27.3	15.5	20.0
	Winter (Dec-Jan-Feb)				*Winter (Dec-Jan-Feb)*				*Winter (Dec-Jan-Feb)*			
CCSM4	8.4	3.5	-7.6	-8.9	43.4	13.6	29.1	29.3	26.1	24.4	45.9	47.4
CNRM-CM5	14.4	15.1	12.7	11.7	33.0	30.1	27.6	28.1	51.8	46.2	40.7	45.8
INM-CM4	43.0	30.7	22.5	24.2	32.5	27.9	23.4	23.7	43.2	30.1	21.0	23.4
HadGEM2-ES	-1.6	-8.4	-1.7	-4.7	17.2	10.2	18.0	14.0	15.9	25.4	16.3	12.3
CanESM2	18.7	30.9	22.7	24.8	61.9	43.9	58.0	60.3	56.7	54.6	50.1	49.8
IPSL-CM5A-MR	37.1	35.2	36.2	38.0	45.6	46.2	46.6	46.6	68.8	61.9	67.5	69.5
Average	20.0	17.8	14.1	14.2	38.9	28.7	33.8	33.7	43.8	40.4	40.2	41.3
	All Year (Jan-Dec)				*All Year (Jan-Dec)*				*All Year (Jan-Dec)*			
CCSM4	-22.1	-8.6	-47.4	-63.1	46.7	4.1	29.9	20.0	31.9	25.2	60.5	56.3
CNRM-CM5	41.8	42.7	25.8	42.0	42.6	40.8	31.7	46.2	125.2	117.5	103.6	140.5
INM-CM4	15.1	14.4	4.5	9.2	-12.8	0.4	-5.5	-0.9	-16.6	-12.9	-26.5	-19.9
HadGEM2-ES	-2.6	18.4	-5.6	-2.8	36.3	48.8	34.4	35.2	28.4	56.3	26.3	30.1
CanESM2	39.6	18.0	37.0	39.9	85.5	44.9	78.1	71.6	98.3	70.1	87.7	86.5
IPSL-CM5A-MR	25.5	22.2	29.1	33.1	-8.3	-8.4	2.0	2.7	65.9	60.6	71.0	71.5
Average	16.2	17.9	7.2	9.7	31.7	21.7	28.4	29.1	55.5	52.8	53.8	60.8

(f) Changes in seasonal and annual precipitation (mm) under RCP8.5 relative to 1961-1990.

Datasets	NEXDCP30	ClimateNA	MACAv2-LIVNEH	MACAv2-METDATA	NEXDCP30	ClimateNA	MACAv2-LIVNEH	MACAv2-METDATA	NEXDCP30	ClimateNA	MACAv2-LIVNEH	MACAv2-METDATA
	2011 - 2040				2041 - 2070				2071 - 2100			
	Spring (Mar-Apr-May)				*Spring (Mar-Apr-May)*				*Spring (Mar-Apr-May)*			
CCSM4	-8.5	2.0	-12.7	-18.4	-13.5	-5.7	-5.8	-9.6	-5.8	-1.7	2.0	4.0
CNRM-CM5	18.9	12.9	13.0	11.6	33.2	28.4	27.9	29.8	14.7	11.5	13.7	14.5
INM-CM4	-8.8	-5.7	-10.2	-9.5	-3.6	-1.8	-4.4	-0.2	33.5	23.2	21.8	35.8
HadGEM2-ES	-3.2	-29.6	-3.0	-3.4	2.2	-34.3	4.8	9.0	-4.4	-41.2	-7.5	-5.7
CanESM2	23.2	7.6	20.7	19.2	35.5	8.0	34.6	36.4	24.0	5.5	23.6	26.4
IPSL-CM5A-MR	-24.2	-21.9	-21.4	-27.0	15.8	14.7	14.1	13.1	-2.5	-4.2	-1.0	-0.8
Average	-0.4	-5.8	-2.3	-4.6	11.6	1.5	11.9	13.1	9.9	-1.2	8.8	12.3
	Summer (Jun-Jul-Aug)				*Summer (Jun-Jul-Aug)*				*Summer (Jun-Jul-Aug)*			
CCSM4	3.3	-4.6	-2.5	-0.2	-11.3	-9.6	-9.3	-12.7	-6.0	-12.1	-15.3	-19.2
CNRM-CM5	-1.4	-1.1	0.7	4.1	-9.6	-8.3	-3.3	3.2	-24.0	-20.5	-14.5	-7.4
INM-CM4	-27.2	-18.3	-16.4	-14.6	-42.1	-30.1	-28.9	-29.9	-47.1	-36.0	-32.7	-33.1
HadGEM2-ES	-16.3	-38.3	-18.5	-18.1	-27.8	-43.1	-31.8	-33.0	-28.6	-48.5	-35.1	-36.4
CanESM2	-12.6	-16.3	-16.6	-19.6	9.1	-6.9	-0.5	-5.3	26.2	3.8	14.7	7.3
IPSL-CM5A-MR	0.1	-0.8	-0.2	5.5	-4.2	-3.9	-4.8	-1.1	-2.1	-1.9	0.8	9.3
Average	-9.0	-13.2	-8.9	-7.1	-14.3	-17.0	-13.1	-13.1	-13.6	-19.2	-13.7	-13.3
	Autumn (Sep-Oct-Nov)				*Autumn (Sep-Oct-Nov)*				*Autumn (Sep-Oct-Nov)*			
CCSM4	27.0	-1.5	2.2	4.3	0.0	-0.8	-5.7	-6.0	17.2	3.1	16.6	16.0
CNRM-CM5	-14.4	-11.8	-9.6	-11.7	33.3	35.3	33.8	38.4	50.9	54.1	49.2	57.8
INM-CM4	-28.3	-20.4	-17.6	-18.5	-23.7	-18.2	-18.6	-16.2	-4.9	-3.8	-4.1	1.0
HadGEM2-ES	39.1	101.2	32.0	36.5	24.5	108.2	26.7	31.1	14.2	126.8	16.3	12.4
CanESM2	-16.1	-3.4	-13.0	-15.3	-5.6	5.3	-3.1	-1.0	1.4	11.0	3.7	4.1
IPSL-CM5A-MR	-0.1	-1.4	1.0	3.7	-1.7	-0.8	1.0	4.4	22.0	22.9	22.2	28.9
Average	1.2	10.5	-0.8	-0.2	4.5	21.5	5.7	8.4	16.8	35.7	17.3	20.1
	Winter (Dec-Jan-Feb)				*Winter (Dec-Jan-Feb)*				*Winter (Dec-Jan-Feb)*			
CCSM4	23.3	3.8	14.4	16.2	53.1	25.5	25.6	24.2	9.5	23.8	54.0	54.2
CNRM-CM5	23.7	21.3	20.9	14.2	49.3	41.6	40.3	38.1	68.6	62.9	63.1	60.3
INM-CM4	38.9	28.4	17.8	17.2	7.0	5.1	6.8	9.6	103.4	77.9	67.6	70.7
HadGEM2-ES	36.3	-7.0	28.1	27.6	16.4	8.2	16.5	17.1	32.2	8.8	29.0	24.8
CanESM2	28.7	41.6	27.1	30.8	48.9	69.7	42.7	43.8	91.5	85.9	82.0	78.2
IPSL-CM5A-MR	51.3	52.6	49.8	54.2	51.9	51.5	53.5	57.2	58.5	58.1	43.9	40.7
Average	33.7	23.4	26.3	26.7	37.7	33.6	30.9	31.7	60.6	52.9	56.6	54.8
	All Year (Jan-Dec)				*All Year (Jan-Dec)*				*All Year (Jan-Dec)*			
CCSM4	45.2	-0.3	1.4	2.0	28.3	9.4	4.8	-4.0	14.9	13.0	57.3	54.9
CNRM-CM5	26.9	21.3	25.0	18.2	106.2	96.9	98.6	109.5	110.2	108.0	111.5	125.3
INM-CM4	-25.4	-15.9	-26.4	-25.4	-62.5	-45.1	-45.1	-36.7	85.0	61.2	52.6	74.4
HadGEM2-ES	55.8	26.4	38.6	42.5	15.2	39.0	16.3	24.2	13.4	46.0	2.8	-4.9
CanESM2	23.1	29.6	18.2	15.1	87.9	76.2	73.8	73.9	143.1	106.3	124.0	116.0
IPSL-CM5A-MR	27.2	28.5	29.1	36.5	61.9	61.5	63.8	73.6	75.9	74.8	66.0	78.2
Average	25.5	14.9	14.3	14.8	39.5	39.6	35.3	40.1	73.7	68.2	69.0	74.0

Supplemental Table S2. Spatially aggregated variability in projected climate change. Standard deviation in projected maximum, minimum temperature and the coefficient of variation in precipitation for the period of 2071-2100 across the four downscaled climate datasets are spatially averaged to each US Geological Survey HUC8 watershed in the Pacific Northwest, USA. The rankings of T_{max}, T_{min} and PPT correspond to the five ranges of values shown in Figure 7a.

HUC	Name	STATES	Area (km^2)	Std_Tmax (°C)	Std_Tmin (°C)	Std_PPT (%)	Tmax_rank	Tmin_rank	PPT_rank
17010307	Lower Spokane	WA	2313.19	0.14	0.24	3	very low	very low	very low
17020003	Colville	WA	2635.25	0.13	1.02	6	very low	medium	low
17020004	Sanpoil	WA	2541.30	0.32	0.99	4	low	medium	very low
17020005	Chief Joseph	WA	3466.99	0.14	0.41	5	very low	very low	very low
17020008	Methow	WA	4710.83	0.35	1.73	9	low	very high	low
17020009	Lake Chelan	WA	2415.06	0.48	1.44	6	medium	high	low
17020010	Upper Columbia-Entiat	WA	3878.88	0.11	0.71	4	very low	low	very low
17020011	Wenatchee	WA	3440.91	0.34	1.33	8	low	high	low
17020012	Moses Coulee	WA	2410.01	0.13	0.25	8	very low	very low	low
17020013	Upper Crab	WA	4810.43	0.16	0.26	3	very low	very low	very low
17020014	Banks Lake	WA	1595.54	0.11	0.12	5	very low	very low	very low
17020015	Lower Crab	WA	6425.13	0.17	0.11	5	very low	very low	very low
17020016	Upper Columbia-Priest Rapids	WA	5415.64	0.16	0.03	4	very low	very low	very low
17030001	Upper Yakima	WA	5539.84	0.24	0.71	3	low	low	very low
17030002	Naches	WA	2860.95	0.25	0.95	2	low	medium	very low
17030003	Lower Yakima	WA	7525.92	0.08	0.44	3	very low	low	very low
17050109	Crooked-Rattlesnake	OR	3442.97	0.12	1.20	3	very low	medium	very low
17050110	Lower Owyhee	OR	5116.40	0.15	0.87	2	very low	medium	very low
17050116	Upper Malheur	OR	6289.16	0.12	0.95	5	very low	medium	very low
17050117	Lower Malheur	OR	2456.58	0.16	0.68	3	very low	low	very low
17050118	Bully	OR	1517.63	0.28	0.45	5	low	low	very low

17050119	Willow	OR	1967.57	0.26	0.45	6	low	low	low
17050202	Burnt	OR	2847.51	0.28	1.13	1	low	medium	very low
17050203	Powder	OR	4423.32	0.19	1.06	2	very low	medium	very low
17060102	Imnaha	OR	2203.18	0.28	1.19	8	low	medium	low
17060104	Upper Grande Ronde	OR	4238.03	0.07	0.80	2	very low	low	very low
17060105	Wallowa	OR	2471.11	0.33	1.22	14	low	high	medium
17060106	Lower Grande Ronde	OR	3932.43	0.10	1.40	7	very low	high	low
17060110	Lower Snake	WA	1876.91	0.11	0.39	8	very low	very low	low
17070101	Middle Columbia-Lake Wallula	OR,WA	6644.46	0.12	0.10	3	very low	very low	very low
17070102	Walla Walla	OR,WA	4600.96	0.41	0.84	3	medium	medium	very low
17070103	Umatilla	OR	6541.54	0.23	0.66	3	low	low	very low
17070104	Willow	OR	2246.61	0.20	0.46	7	very low	low	low
17070105	Middle Columbia-Hood	OR,WA	5589.17	0.08	0.72	4	very low	low	very low
17070106	Klickitat	WA	3501.54	0.13	0.68	4	very low	low	very low
17070201	Upper John Day	OR	5540.17	0.07	1.53	7	very low	high	low
17070202	North Fork John Day	OR	4787.33	0.22	1.70	4	low	very high	very low
17070203	Middle Fork John Day	OR	2051.80	0.13	1.59	4	very low	high	very low
17070204	Lower John Day	OR	8155.46	0.12	0.67	5	very low	low	very low
17070301	Upper Deschutes	OR	5579.64	0.10	0.64	10	very low	low	low
17070302	Little Deschutes	OR	2727.07	0.10	0.64	6	very low	low	low
17070303	Beaver-South Fork	OR	3963.48	0.06	0.43	4	very low	low	very low
17070304	Upper Crooked	OR	2993.89	0.20	1.18	7	very low	medium	low
17070305	Lower Crooked	OR	4786.96	0.10	0.86	4	very low	medium	very low
17070306	Lower Deschutes	OR	5945.94	0.14	0.49	6	very low	low	low
17070307	Trout	OR	1792.70	0.13	0.95	5	very low	medium	very low
17080001	Lower Columbia-Sandy	OR,WA	2264.58	0.23	0.56	8	low	low	low

17080002	Lewis	WA	2720.88	0.06	0.92	12	very low	medium	medium
17080003	Lower Columbia-Clatskanie	OR,WA	2964.24	0.17	0.32	7	very low	very low	low
17080004	Upper Cowlitz	WA	2655.27	0.19	1.01	4	very low	medium	very low
17080005	Lower Cowlitz	WA	3758.78	0.26	0.56	3	low	low	very low
17080006	Lower Columbia	OR,WA	1754.06	0.53	0.54	5	medium	low	very low
17090001	Middle Fork Willamette	OR	3540.38	0.66	1.22	3	medium	high	very low
17090002	Coast Fork Willamette	OR	1726.13	0.75	1.23	4	high	high	very low
17090003	Upper Willamette	OR	4850.08	0.17	0.38	3	very low	very low	very low
17090004	Mckenzie	OR	3468.20	0.52	1.14	4	medium	medium	very low
17090005	North Santiam	OR	1979.32	0.42	0.86	2	medium	medium	very low
17090006	South Santiam	OR	2696.14	0.47	0.94	3	medium	medium	very low
17090007	Middle Willamette	OR	1841.44	0.07	0.22	3	very low	very low	very low
17090008	Yamhill	OR	1998.59	0.12	0.28	7	very low	very low	low
17090009	Molalla-Pudding	OR	2267.49	0.20	0.41	3	very low	very low	very low
17090010	Tualatin	OR	1835.90	0.23	0.24	2	low	very low	very low
17090011	Clackamas	OR	2441.60	0.60	0.83	2	medium	medium	very low
17090012	Lower Willamette	OR	1053.18	0.03	0.14	4	very low	very low	very low
17100103	Upper Chehalis	WA	3362.41	0.23	0.38	5	low	very low	very low
17100104	Lower Chehalis	WA	2118.55	0.22	0.35	7	low	very low	low
17100105	Grays Harbor	WA	1520.73	0.43	0.36	9	medium	very low	low
17100202	Nehalem	OR	2213.54	0.46	0.58	5	medium	low	very low
17100206	Siuslaw	OR	2007.91	0.36	0.59	3	low	low	very low
17100301	North Umpqua	OR	3558.42	0.63	0.98	3	medium	medium	very low
17100302	South Umpqua	OR	4665.86	0.46	0.73	6	medium	low	low
17100303	Umpqua	OR	3921.40	0.23	0.55	3	low	low	very low
17100305	Coquille	OR	2737.21	0.52	0.88	5	medium	medium	very low

17100307	Upper Rogue	OR	4183.05	0.13	0.84	3	very low	medium	very low
17100308	Middle Rogue	OR	2284.46	0.11	0.81	5	very low	medium	very low
17100310	Lower Rogue	OR	2348.77	0.49	0.53	5	medium	low	very low
17110006	Sauk	WA	1896.85	0.73	1.51	5	high	high	very low
17110007	Lower Skagit	WA	1172.40	0.44	0.70	10	medium	low	low
17110008	Stillaguamish	WA	1818.76	0.34	1.03	13	low	medium	medium
17110009	Skykomish	WA	2161.39	0.46	0.99	10	medium	medium	low
17110010	Snoqualmie	WA	1797.19	0.35	0.75	7	low	low	low
17110011	Snohomish	WA	755.25	0.07	0.38	8	very low	very low	low
17110012	Lake Washington	WA	1572.01	0.16	0.30	3	very low	very low	very low
17110013	Duwamish	WA	1257.91	0.26	0.38	1	low	very low	very low
17110014	Puyallup	WA	2550.65	0.61	0.84	10	medium	medium	low
17110015	Nisqually	WA	1993.99	0.11	0.31	7	very low	very low	low
17110016	Deschutes	WA	442.17	0.31	0.51	16	low	low	high
17110017	Skokomish	WA	634.46	0.42	1.09	4	medium	medium	very low
17110018	Hood Canal	WA	2459.07	0.63	1.70	4	medium	very high	very low
17110019	Puget Sound	WA	6353.55	0.09	0.28	2	very low	very low	very low
17120001	Harney-Malheur Lakes	OR	3761.88	0.19	0.46	7	very low	low	low
17120002	Silvies	OR	3414.34	0.18	0.61	4	very low	low	very low
17120003	Donner und Blitzen	OR	2045.47	0.13	1.15	3	very low	medium	very low
17120004	Silver	OR	4361.21	0.11	0.07	5	very low	very low	very low
17120005	Summer Lake	OR	10708.36	0.13	0.17	4	very low	very low	very low
17120006	Lake Abert	OR	2670.81	0.06	0.38	9	very low	very low	low
18010201	Williamson	OR	3725.89	0.14	0.25	5	very low	very low	very low
18010202	Sprague	OR	4170.89	0.17	0.59	3	very low	low	very low
18010203	Upper Klamath Lake	OR	1875.14	0.18	0.40	4	very low	very low	very low
